# EMT-related gene risk model establishment for prognosis and drug treatment efficiency prediction in hepatocellular carcinoma

**DOI:** 10.1038/s41598-023-47886-z

**Published:** 2023-11-21

**Authors:** Xiaqing Gao, Chunting Yang, Hailong Li, Lihua Shao, Meng Wang, Rong Su

**Affiliations:** 1 The First Clinical Medical College, Gansu University of Chinese Medicine, Lanzhou, 730000 Gansu People’s Republic of China; 2 Department of Geriatrics, Affiliated Hospital of Gansu University of Chinese Medicine, Lanzhou, 730000 Gansu People’s Republic of China; 3 Key Laboratory of Traditional Chinese Herbs and Prescription Innovation and Transformation of Gansu Province and Gansu Provincial Traditional Chinese Medicine New Product Innovation Engineering Laboratory, Gansu University of Chinese Medicine, Lanzhou, 730000 Gansu People’s Republic of China; 4 Key Laboratory of Dunhuang Medicine and Transformation, Ministry of Education, Gansu University of Chinese Medicine, Lanzhou, 730000 Gansu People’s Republic of China

**Keywords:** Cancer, Biomarkers

## Abstract

This study was designed to evaluate the prognosis and pharmacological therapy sensitivity of epithelial mesenchymal transition-related genes (EMTRGs) that obtained from the EMTome database in hepatocellular carcinoma (HCC) using bioinformatical method. The expression status of EMTRGs were also investigated using the clinical information of HCC patients supported by TCGA database and the ICGC database to establish the TCGA cohort as the training set and the ICGC cohort as the validation set. Analyze the EMTRGs between HCC tissue and liver tissue in the TCGA cohort in the order of univariate COX regression, LASSO regression, and multivariate COX regression, and construct a risk model for EMTRGs. In addition, enrichment pathways, gene mutation status, immune infiltration, and response to drugs were also analyzed in the high-risk and low-risk groups of the TCGA cohort, and the protein expression status of EMTRGs was verified. The results showed a total of 286 differentially expressed EMTRGs in the TCGA cohort, and EZH2, S100A9, TNFRSF11B, SPINK5, and CCL21 were used for modeling. The TCGA cohort was found to have a worse outcome in the high-risk group of HCC patients, and the ICGC cohort confirmed this finding. In addition, EMTRGs risk score was shown to be an independent prognostic factor in both cohorts by univariate and multivariate COX regression. The results of GSEA analysis showed that most of the enriched pathways in the high-risk group were associated with tumor, and the pathways enriched in the low-risk group were mainly associated with metabolism. Patients in various risk groups had varying immunological conditions, and the high-risk group might benefit more from targeted treatments. To sum up, the EMTRGs risk model was developed to forecast the prognosis for HCC patients, and the model might be useful in assisting in the choice of treatment drugs for HCC patients.

## Introduction

Liver cancer is one of the malignant tumors with the highest incidence worldwide. Epidemiological data in 2022 showed that there were about 430,000 liver cancer cases and 300,000 liver cancer deaths in China, and its incidence and mortality rate were in the fourth and second place of malignant tumors, respectively^1^. The most effective therapies for liver cancer are surgical resection, ablation, liver transplantation, radiation, and medication, but these procedures are only useful for people with early-stage who have not yet developed into metastasis, and they also have adverse effects^2^. Due to the insidious onset of liver cancer and the lack of specific and sensitive molecular markers for diagnosis, most patients are already in an advanced stage when they are diagnosed and have distant metastasis, resulting in a very low 5-year survival rate^3–5^. An in-depth exploration of biomarkers in the development and metastasis of liver cancer will help establish new diagnostic and therapeutic approaches.

Metastasis is the cause of death in 90% of cancer patients, and tumors can acquire characteristics such as migration and invasion, anti-apoptosis, and immune tolerance during EMT^6^. The concept of epithelial mesenchymal transition (EMT) was first introduced in the field of embryology in 1982, when researchers discovered that lens epithelial cells could form pseudopods in collagen gels and transform into mesenchymal cell-like forms^7^. The process of EMT refers to the dynamic change of cellular tissue from epithelial to mesenchymal phenotypes, which in turn leads to changes in cell migration and invasive capacity^8^. According to previously published studies, EMT closely correlates with tumor invasion and metastasis, primarily through downregulating epithelial markers and upregulating mesenchymal markers, which results in reduced cell adhesion and increased cell mobility and encourages tumor invasion and metastasis^9^. EMT is regarded to be a fundamental regulator of treatment resistance, assisting tumor cells to avoid being destroyed by the immune system, in addition to promoting growth and metastasis^10,11^. The study of EMT in enhancing cancer metastasis, drug resistance, and leading to poor patient prognosis has been widely confirmed. It was discovered that HCC could generate more exosomes in hypoxic conditions, and that these exosomes contained the miRNA-273f. that may cause EMT in HCC cells by triggering the Wnt/-catenin pathway, dramatically increasing cell invasion and metastasis^12^. Using tissue samples from HCC patients, researchers discovered that the cancer gene PRMT9 might cause EMT via the phosphate PI3K/AKT/GSK-3/Snail signal pathway, boosting snail expression and quickening the remote transmission of HCC^13^. In addition, inhibition of EMT can increase the sensitivity of HCC to chemotherapy, as in epidermal growth factor receptor targeted therapy, epithelial cells are more sensitive than stromal cells^14^. It has also been confirmed that using siRNA interference to reduce the expression of Long non coding RNA H19 can reverse the EMT process of HCC and improve the sensitivity of patients to sorafenib using immunofluorescence experiments^14^. Qi et al. found that EMT can induce the release of circulating tumor cells in HCC patients, which are important markers of recurrence and poor prognosis. Patients with higher levels of EMT have lower five-year survival rates^15^. Studies of EMTRGs predictor markers based on open-access databases like TCGA, ICGC, and GEO have been thoroughly studied in recent years in a variety of malignant tumors, including pancreatic cancer^16,17^, prostate cancer^18^, colon cancer^19,20^, gastric cancer^21,22^ and endometrial cancer^23,24^. Therefore, searching for biomarkers targeting EMT and intervening in the EMT process can help suppress tumor metastasis and increase the sensitivity of tumor cells to anticancer drugs, thereby improving the survival rate and quality of life of cancer patients.

Based on the TCGA database, we screened differentially expressed EMTRGs and constructed a risk score model, which in turn came to mine the prognostic characteristics of new EMTRGs for HCC. The purpose of this study is to investigate new prognostic indicators for the diagnosis and treatment of HCC patients as well as to reveal prospective therapeutic targets and new insights into the mechanism and function of EMT in the development of HCC.

## Material and methods

### Data collection

UCSC Xena (http://xena.ucsc.edu/) is a derivative website for secondary development of TCGA data, supporting data analysis functions and visualization^25^. RNAseq gene expression profiles and clinical follow-up information of TCGA-LIHC patients were obtained from UCSC Xena as a training dataset. In addition, International Cancer Genome Consortium (ICGC) (https://dcc.icgc.org/) is a non-profit international organization aimed at providing data and resource support for global cancer genomics research, with samples from different countries and regions. RNAseq gene expression profiles and clinical follow-up information of LIRI-JP patients were obtained from ICGC database as the validation dataset, and ICGC samples were mainly derived from Japanese people infected with the hepatitis virus. The samples without clinical information and expression profiles in the two cohorts were deleted, and the clinical pathological parameters such as age, gender, TNM stage, grade, etc. were obtained. In this study, we screened differentially expressed genes based on the data from the TCGA cohort. The mRNA expression data and exact clinical characteristics, including survival time and tumor characteristics, were then analyzed for all patients in both cohorts. EMTRGs were obtained from EMTome (http://www.emtome.org/)^26^.

### Screening of differentially expressed genes in EMT

The mRNA expression profiles of 50 liver tissues and 373 HCC tissues from the TCGA cohort were screened for differential genes using the Limma software package^27^. Differential genes were intersected with EMTRGs by jvenn (https://jvenn.toulouse.inrae.fr/app/index.html ) to obtain EMT differentially expressed genes^28^.

### Construction and validation of EMTRGs risk model

A total of 365 mRNA expression data with overall survival information were retrieved after normal samples and samples with unknown survival information were eliminated from the TCGA cohort's mRNA expression data. TCGA cohorts were analyzed in the order of univariate COX regression, LASSO regression and multivariate COX regression through the survival software package and glmnet software package. The expression values and coefficients of genes were then used to build risk models. According to the modeling equations, the risk scores of each patient in the TCGA cohort and the ICGC cohort were determined, and high-risk and low-risk groups were determined based on the median risk score. The survival software package examined the prognostic differences between the high-risk and low-risk groups. The pROC software package were then used to execute ROC curve analysis to generate AUC values and test the efficacy of the risk model in predicting prognosis. Simultaneous univariate and multivariate COX regression methods were used to assess the role of risk score in overall survival in two cohorts. Based on risk scores from the TCGA cohort, a nomogram was created using the rms software package, and a calibration curve was used to predict survival at 365, 1095, and 1825 days for patients with HCC. Finally, the relationship between high-risk and low-risk groups HCC patients' prognoses and various clinical and pathological characteristics were examined.

### GSEA and gene mutation analysis

To explore its biosignal pathways, GSEA software (v3.0) was obtained from the GSEA website (https://www.gsea-msigdb.org/gsea/index.jsp)^29^, the samples were divided into high-risk and low-risk groups based on risk scores, and the c2.cp.kegg.v7.4.symbols.gmt subset were downloaded from the Molecular Signatures Database to evaluate the pathways and molecular mechanisms. Based on gene expression spectrum and grouping, the geneset ranges from the minimum set of genes of 5 to the maximum set of genes of 5000 was established to resample for a thousand times, and the Kyoto Encyclopedia of Genes and Genomes (KEGG) enrichment pathway were performed and selected based on *P* value < 0.05 and FDR value < 0.25. Additionally, the online database SangerBox 3.0 (http://vip.sangerbox.com/home.html) was used to assess the mutation landscapes of the top 15 genes with the greatest mutation frequency in the TCGA cohort in the high-risk and low-risk groups^30^.

### Immune cell infiltration degree and immune subtype analysis

To explore the relationship between risk score and prognosis of HCC, risk score and tumor microenvironment were analyzed. The ESTIMATE (https://bioinformatics.mdanderson.org/estimate/) website could provide and analyze the data about Stromal Scores, Immune Scores, and ESTIMATE Scores of HCC patients based on expression data, and make them visualized. The infiltration of different types of immune cells in the HCC microenvironment was assessed using the CIBERSORT algorithm in the immunedeconv software package. Finally, the immune subtypes of patients with HCC in TCGA Pan-Cancer were obtained from UCSC Xena.

### Drug sensitivity prediction

To understand the therapeutic response to different chemotherapeutic agents in the high-risk and low-risk groups, the chemotherapeutic response of each sample was predicted based on Genomics of Cancer Drug Sensitivity (GDSC), a process that consists of the pRRophetic software package were then used to compare the half maximal inhibitory concentration (IC50) of commonly used chemotherapeutic agents between the two groups.

### Protein expression analysis and statistical analysis

UALCAN (https://ualcan.path.uab.edu/index.html) is a comprehensive, user-friendly, and interactive web resource for analyzing cancer OMICS data^31^. Protein expression in the EMTRGs model was examined using the CPTAC cohort from the UALCAN database. Additionally, the Human Protein Atlas database (https://www.proteinatlas.org/) utilizes transcriptomics and proteomics techniques to study protein expression in different tissues and organs at the RNA and protein levels^32^. The Human Protein Atlas database compared the levels of protein expression in HCC and liver tissue. The independent t-test was used for continuous variables, such as Immune score and matrix score. The wilcoxon test was used for Cibesort immunity score, IC50, Mann–Whitney U test was used for Non parametric hypothesis testing methods, and a log-rank test was used for K–M curves to calculate survival differences. A *p*-value of 0.05 indicates that the differences are statistically significant.

## Results

### Variance analysis of EMTRGs

**Figure 1:**
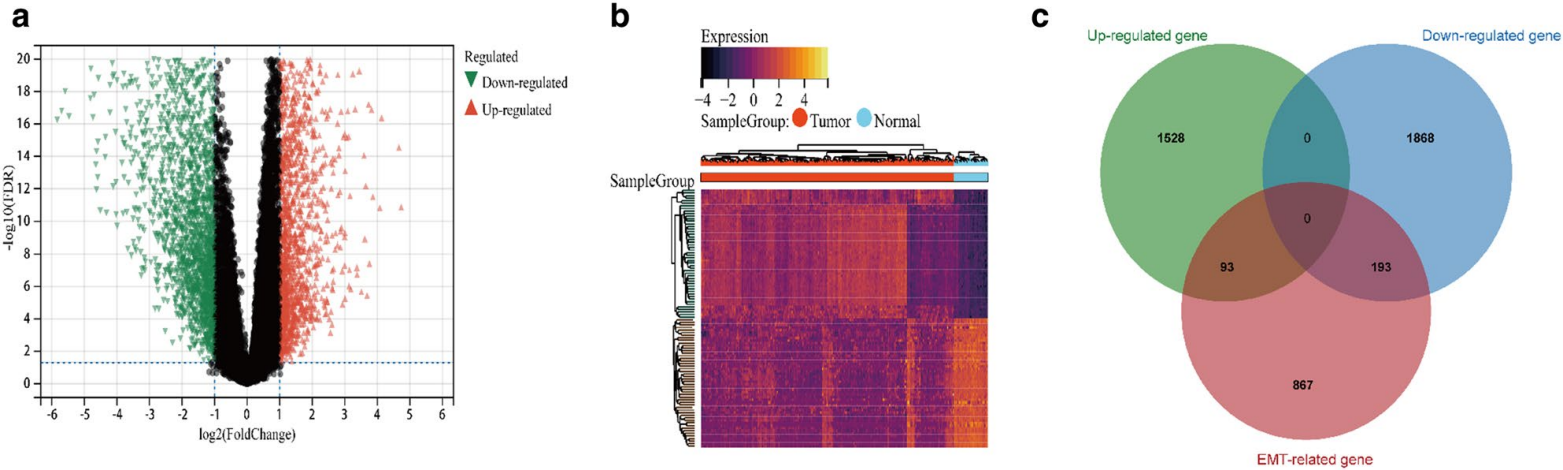
EMTRGs analysis of HCC patients. (**a**) Volcano plot: Red represents up-regulated genes, green represents down-regulated genes, and black represents non-significant genes. (**b**) Heat map of differentially expressed genes. (**c**) Venn diagram: Intersection of differential expression genes with EMTRGs.

Detailed features of the TCGA cohort are summarized in Table 1. A total of 1153 EMTRGs were obtained from the EMTome database. Based on the criteria of | log2 (fold change) |> 2 and FDR < 0.05, differential expression analysis was performed on the TCGA cohort, resulting in 3685 differentially expressed genes. Among them, 1621 genes were up-regulated and 2061 genes were down-regulated compared to normal liver tissue. The above mentioned differentially expressed genes were then intersected with EMTRGs taken to get 286 EMT differentially expressed genes (Fig. 1c).

**Table 1:** Detailed characteristics of the TCGA cohort.

Characteristics	Alive (N = 245)	Dead (N = 132)	Total (N = 377)
Gender			
Female	71 (18.83%)	51 (13.53%)	122 (32.36%)
Male	174 (46.15%)	81 (21.49%)	255 (67.64%)
Age			
Mean ± SD	58.21 ± 13.25	61.76 ± 13.73	59.45 ± 13.51
Median [min–max]	60.00 [16.00,84.00]	64.00 [18.00,90.00]	61.00 [16.00,90.00]
Weight			
Mean ± SD	73.43 ± 20.32	71.88 ± 17.62	72.89 ± 19.41
Median[min–max]	69.00 [40.00,172.00]	69.00 [40.00,139.00]	69.00 [40.00,172.00]
Histological type			
Fibrolamellar Carcinoma	3 (0.80%)	0 (0%)	3 (0.80%)
Hepatocellular Carcinoma	236 (62.60%)	131 (34.75%)	367 (97.35%)
Hepatocholangiocarcinoma (Mixed)	6 (1.59%)	1 (0.27%)	7 (1.86%)
Grade			
G1	37 (9.95%)	18 (4.84%)	55 (14.78%)
G2	118 (31.72%)	62 (16.67%)	180 (48.39%)
G3	81 (21.77%)	43 (11.56%)	124 (33.33%)
G4	8 (2.15%)	5 (1.34%)	13 (3.49%)
Stage			
Stage I	131 (37.11%)	44 (12.46%)	175 (49.58%)
Stage II	61 (17.28%)	26 (7.37%)	87 (24.65%)
Stage III	41 (11.61%)	45 (12.75%)	86 (24.36%)
Stage IV	2 (0.57%)	3 (0.85%)	5 (1.42%)

### EMTRGs risk models can predict the prognosis for HCC patients

**Figure 2:**
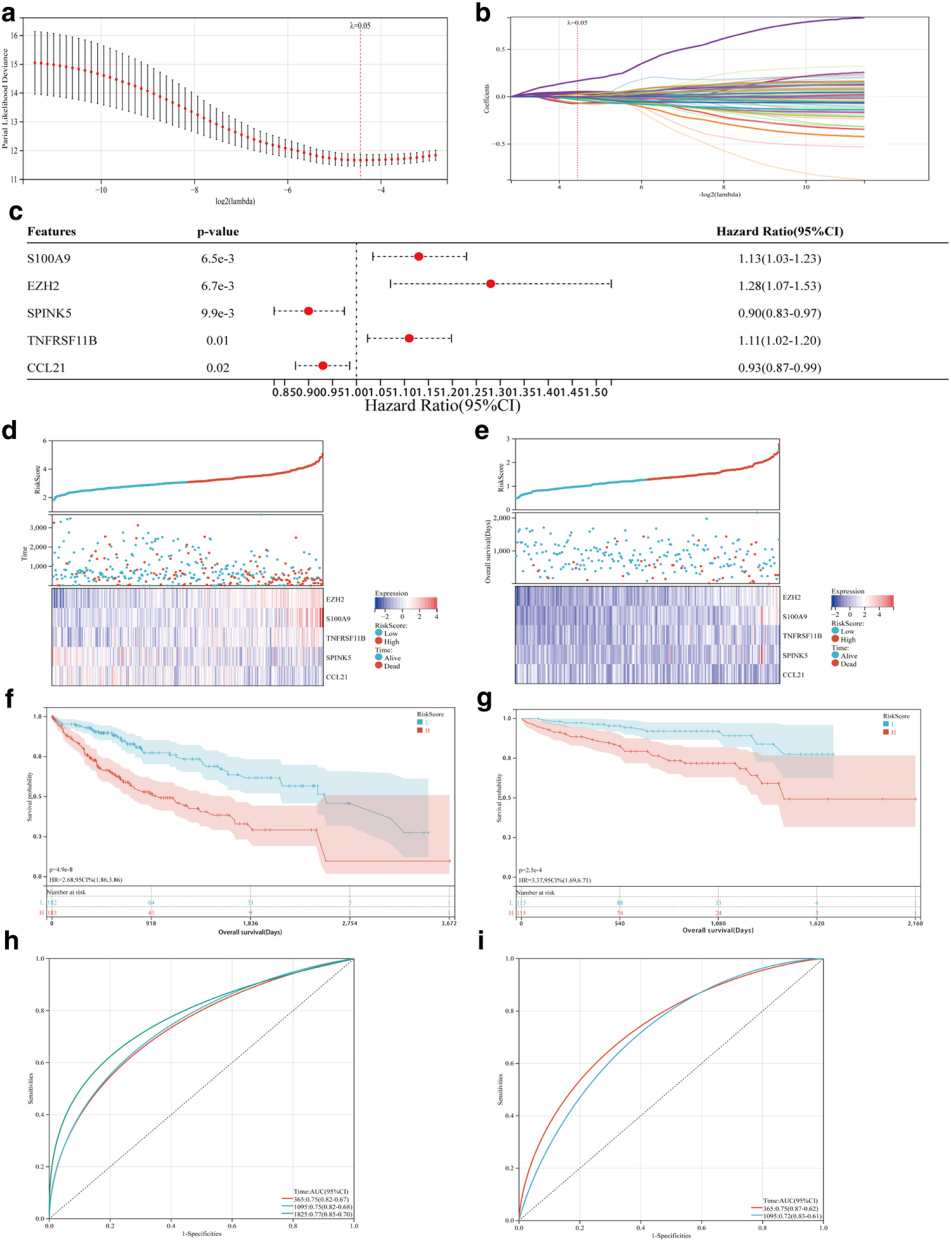
The predictive characteristics of EMTRGs was established to predict the specific survival of HCC patients. (**a**) Penalty maps for EMTRGs LASSO models in HCC. (**b**) LASSO coefficient mapping of EMTRGs. (**c**) Multivariate COX regression gets 5 EMTRGs. (**d**) The heat map of the TCGA cohort. (**e**) The heat map of the ICGC cohort. (**f**) Survival curve chart of the TCGA cohort. (**g**) Survival curve chart of the ICGC cohort. (**h**) ROC curve chart of the TCGA cohort. (**i**) ROC curve chart of the ICGC cohort.

A total of 78 genes out of the 286 EMT differentially expressed genes were connected to analysis the overall survival of HCC patients (Supplementary Fig. 1). LASSO regression analysis was used to screen 17 of these 78 genes (Fig. 2a,b). Finally, multivariate COX regression was utilized to identify five EMTRGs (EZH2, S100A9, TNFRSF11B, SPINK5, CCL21) as independent risk variables among these 17 genes. These EMTRGs were then used to build an EMTRGs risk model (Fig. 2c). EMTRGs risk score formula was as follows : RiskScore = 0.320889050455719 * EZH2 + 0.144247872403908 * S100A9 + 0.079363749195301 * TNFRSF11B − 0.0968623157103071 * SPINK5-0.0819194509885345 * CCL21. Patients with HCC in the two cohorts had risk scores were calculated, and based on the median risk score value for each cohort, patients were split into high-risk and low-risk groups. The prognostic heat map and K-M curves showed that patients in the high-risk group had poorer outcomes in both of these 2 cohorts, the TCGA cohort and the ICGC cohort, and the ROC curve results for the 2 cohorts showed that the risk score model had AUC values greater than 0.7 at 365, 1095, and 1825 days, indicating that the risk score model had good predictive performance for HCC patients (Fig. 2d–i).

**Figure 3:**
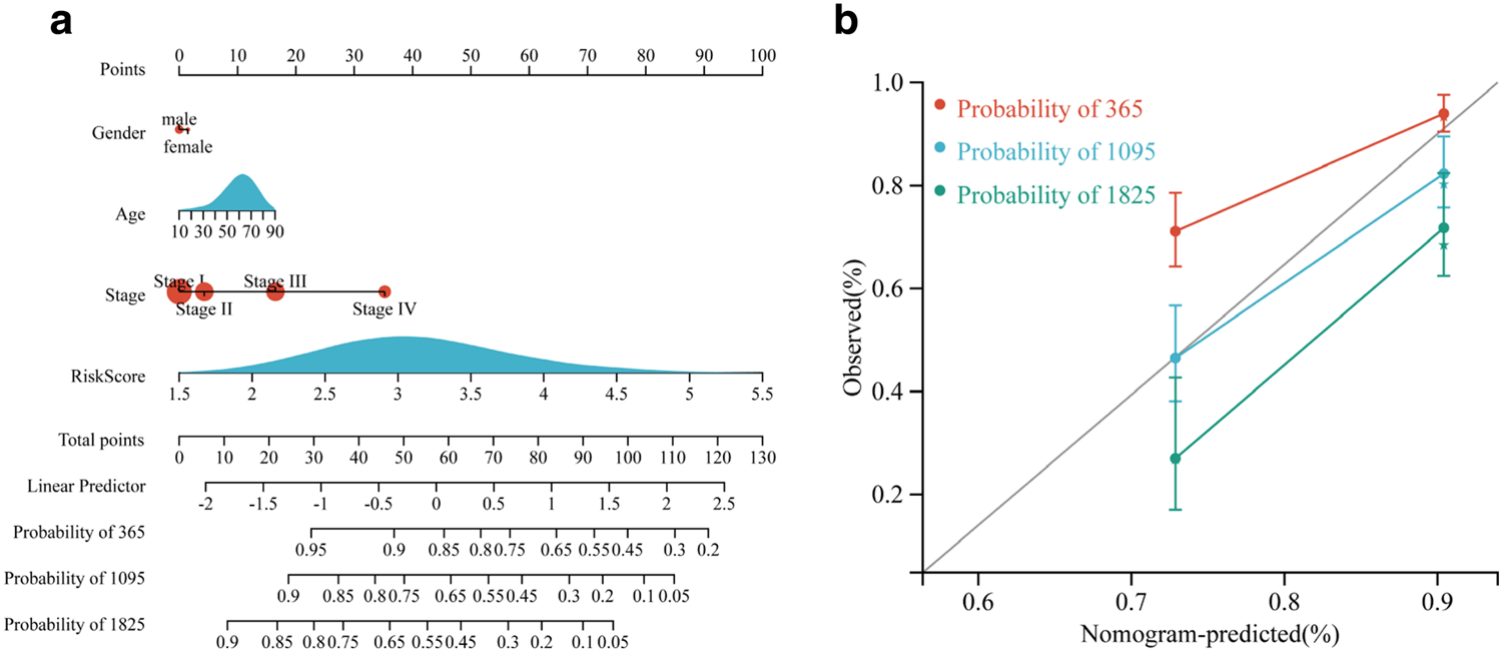
Construction of the nomogram and calibration curve for the TCGA cohort. **(a**) Construction of the nomogram based on gender, age, stage, and risk score of HCC patients. (**b**) The accuracy of nomogram in predicting the survival of patients at 365, 1095, and 1825 days was assessed with a calibration curve.

In these 2 cohorts, EMTRGs risk score was demonstrated to be an independent predictive factor for patients with HCC (Table 2). In order to predict the overall survival of HCC patients, a nomogram (Fig. 3a) and calibration curve (Fig. 3b) incorporating the patient's gender, age, stage, and risk score of EMTRGs were also built.

**Table 2:** Univariate and Multivariate COX regressions for the TCGA cohort and the ICGC cohort.

Variables	Univariate COX regression		Multivariate COX regression	
	HR(95%CI)	*p*-value	HR(95%CI)	*p*-value
TCGA cohort				
Gender		0.26		
Male	Reference			
Female	1.225 (0.860–1.746)	0.26		
Age	1.012 (0.999–1.026)	0.078	1.008 (0.994–1.023)	0.269
Stage		**< 0.001**		
Stage I	Reference			
Stage II	1.423 (0.872–2.323)	0.158	1.185 (0.722–1.944)	0.502
Stage III	2.676 (1.754–4.083)	**< 0.001**	1.902 (1.221–2.963)	**0.004**
Stage IV	5.496 (1.695–17.821)	**0.005**	4.066 (1.250–13.226)	**0.02**
Risk Score	2.986 (2.254–3.956)	**< 0.001**	2.653 (1.940–3.629)	**< 0.001**
ICGC cohort				
Age	1.004 (0.974–1.036)	0.792		
Gender		0.034		
Male	Reference			
Female	1.974 (1.054–3.696)	**0.034**	2.707 (1.394–5.257)	0.003
Stage		**< 0.001**		
Stage II	Reference			
Stage I	0.169 (0.022–1.268)	0.084	0.160 (0.021–1.201)	0.075
Stage III	1.502 (0.749–3.013)	0.252	1.732 (0.838–3.580)	0.138
Stage IV	4.396 (1.947–9.927)	**< 0.001**	2.958 (1.169–7.487)	**0.022**
Risk Score	3.446 (1.853–6.409)	**< 0.001**	2.507 (1.277–4.920)	**0.008**

**Figure 4:**
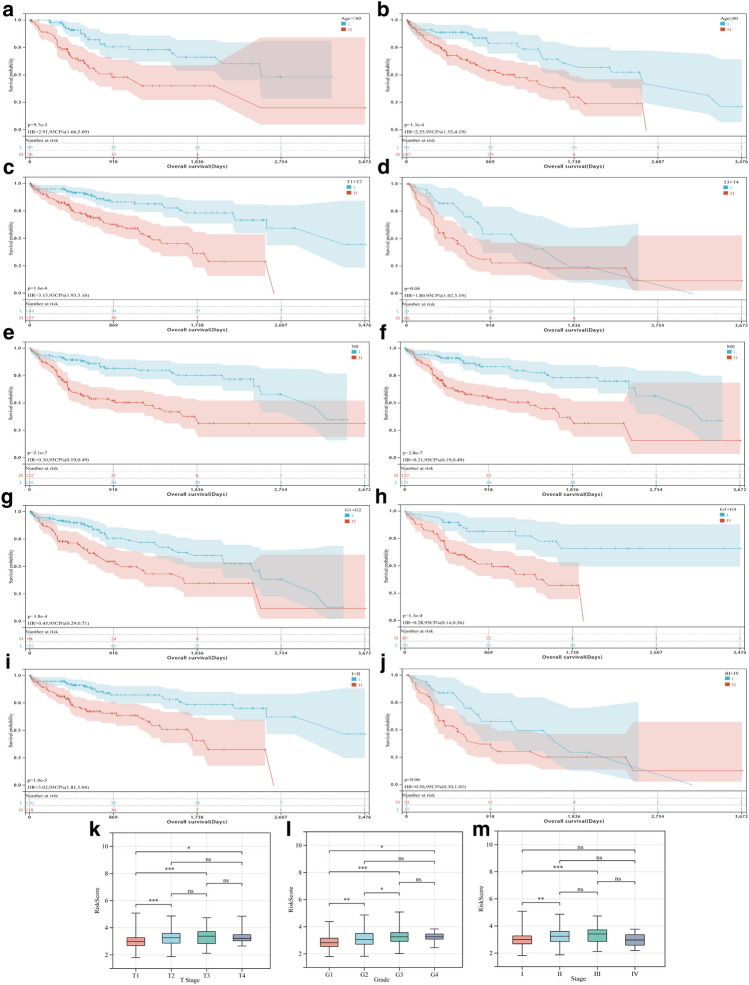
Relationship between high-risk and low-risk groups with different clinicopathological characteristics and prognostic performance. (**a**) Prognostic analysis of HCC patients with Age < 60. (**b**) Prognostic analysis of HCC patients with Age ≥ 60. (**c**) Prognostic analysis of HCC patients with T1 + T2 stages. (**d**) Prognostic analysis of HCC patients with T3 + T4 stages. (**e**) Prognostic analysis of HCC patients with N0 stage. (**f**) Prognostic analysis of HCC patients with M0 stage. (**g**) Prognostic analysis of HCC patients with G1 + G2 stages. (**h**) Prognostic analysis of HCC patients with G3 + G4 stages. (**i**) Prognostic analysis of HCC patients with Stages I + II. **(j**) Prognostic analysis of HCC patients with Stages III + IV. **k** Relationship between T Stage and risk score. (**l**) Relationship between Grade and risk score. (**m**) Relationship between Stage and risk score. (*P < 0.05, **P < 0.01, ***P < 0.001, ns represents no statistical significance).

In the analysis of different risk groups with clinicopathological characteristics, it was found that the prognosis was worse in the high-risk group compared with the low-risk group (Fig. 4a–i), and the survival of Stage III + Stage IV was not statistically significant (Fig. 4j). The risk score increased significantly with increasing T stage, grade, and stage, except for Stage IV (Fig. 4k–m).

### GSEA and gene mutation analysis

**Figure 5:**
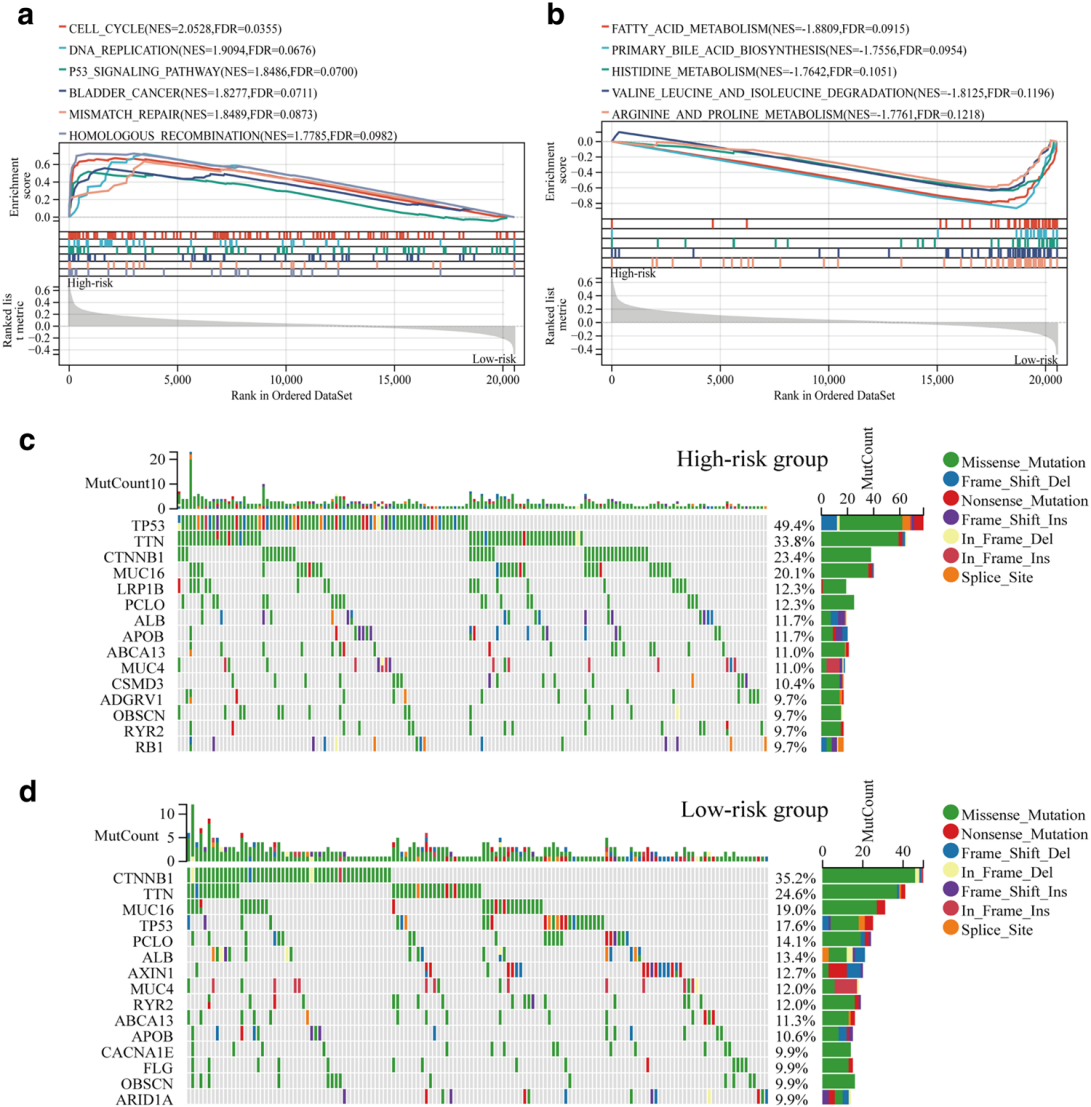
Analysis of GSEA and gene mutation results. (**a**) Major pathway of enrichment in the high-risk group. (**b**) Major pathways of enrichment in the low-risk group. (**c**) Significantly mutated genes in the high-risk group in the TCGA cohort. (**d**) Significantly mutated genes in the low-risk group in the TCGA cohort.

This study examined GSEA for both high-risk and low-risk groups, taking into account the fact that risk scores of HCC patients are negatively correlated with prognosis. The results showed that the main enrichment pathways in the high-risk group included "KEGG_CELL_CYCLE", "KEGG_DNA_REPLICATION","KEGG_P53_SIGNALING_PATHWAY","KEGG_BLADDER_CANCER", and "KEGG_MISMATCH_REPAIR" and " KEGG_HOMOLOGOUS_RECOMBINATION" (Fig. 5a). The main enrichment pathways in the low-risk group including "KEGG_FATTY_ACID_METABOLISM", "KEGG_PRIMARY_BILE_ACID_BIOSYNTHESIS", "KEGG_HISTIDINE_METABOLISM","KEGG_VALINE_LEUCINE_AND_ISOLEUCINE_DEGRADATION" and "KEGG_VALINE_LEUCINE_DEGRADATION" (Fig. 5b). Most of the enriched pathways in the high-risk group were tumor-related, and the enriched pathways in the low-risk group were mainly metabolism-related, which demonstrated the accuracy of predicting a poorer prognosis in the high-risk group in the model were constructed.

The TCGA cohort's high-risk and low-risk groups underwent gene mutation analysis. A series of TP53, TTN, CTNNB1, MUC16, PCLO, ALB, APOB, ABCA13, and MUC4 were discovered to have mutation frequencies greater than 10% in both groups when the top 15 genes with the greatest mutation frequencies were examined. In comparison to the low-risk group, the frequency of TP53 mutations was noticeably higher in the high-risk group (Fig. 5c,d).

### Patients in different risk groups exhibit different immune status

**Figure 6:**
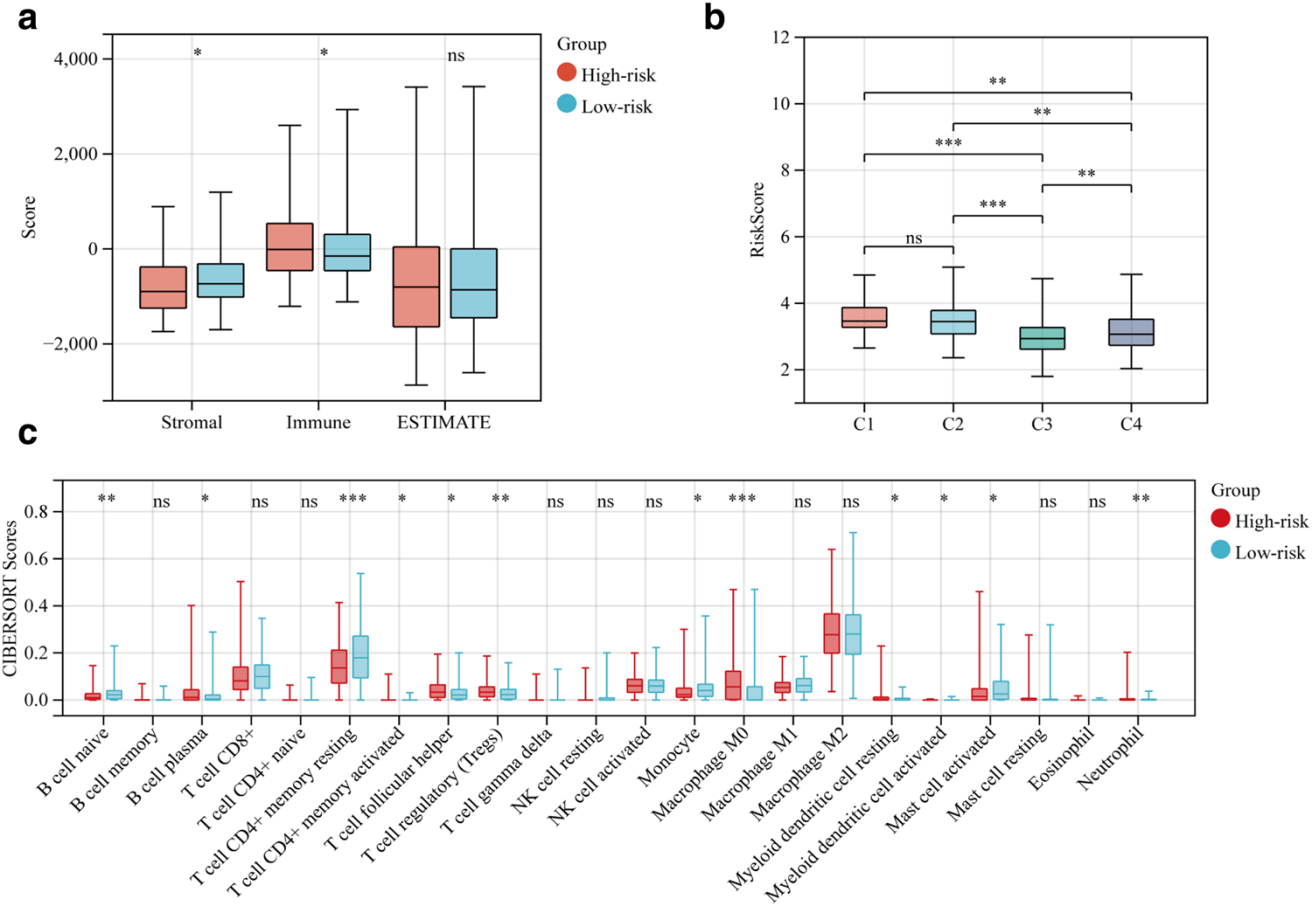
Different risk groups associated with the tumor immune microenvironment. (**a**) ESTIMATE algorithm to calculate Stromal score, Immune score, and ESTIMATE between high-risk and low-risk groups. (**b**) Comparison of different immune subtypes with risk scores. (**c**) CIBERSORT algorithm to calculate cell infiltration level between high-risk and low-risk groups. (*P < 0.05, **P < 0.01, ***P < 0.001, ns represents no statistical significance).

The ESTIMATE algorithm showed that while there was no discernible difference in ESTIMATE score between the two groups, stromal score was lower and immune score was greater in the high-risk group compared to the low-risk group (Fig. 6a). There were 12 clusters of immune cell levels that differed between the high-risk and low-risk groups. The infiltration levels of B cell plasma, T cell CD4^+^ memory activated, T cell follicular helper, T cell regulatory (Tregs), Macrophage M0, Myeloid dendritic cell resting, and neutrophil were found to be higher in the high-risk group than in the low-risk group by the CIBERSORT algorithm (Fig. 6c).

The TCGA database classifies tumors into six subtypes based on immune status: Wound Healing (Immune C1), IFN-gamma Dominant (Immune C2), Inflammatory (Immune C3), Lymphocyte Depleted (Immune C4), Immunologically Quiet (Immune C5), and TGF-beta Dominant (Immune C6). Higher risk scores were strongly related with C1 subtypes, while lower risk scores were significantly associated with C3 subtypes, according to immunosubtyping research (Fig. 6b).

### EMTRGs risk model can be used for treatment strategy selection

**Figure 7:**
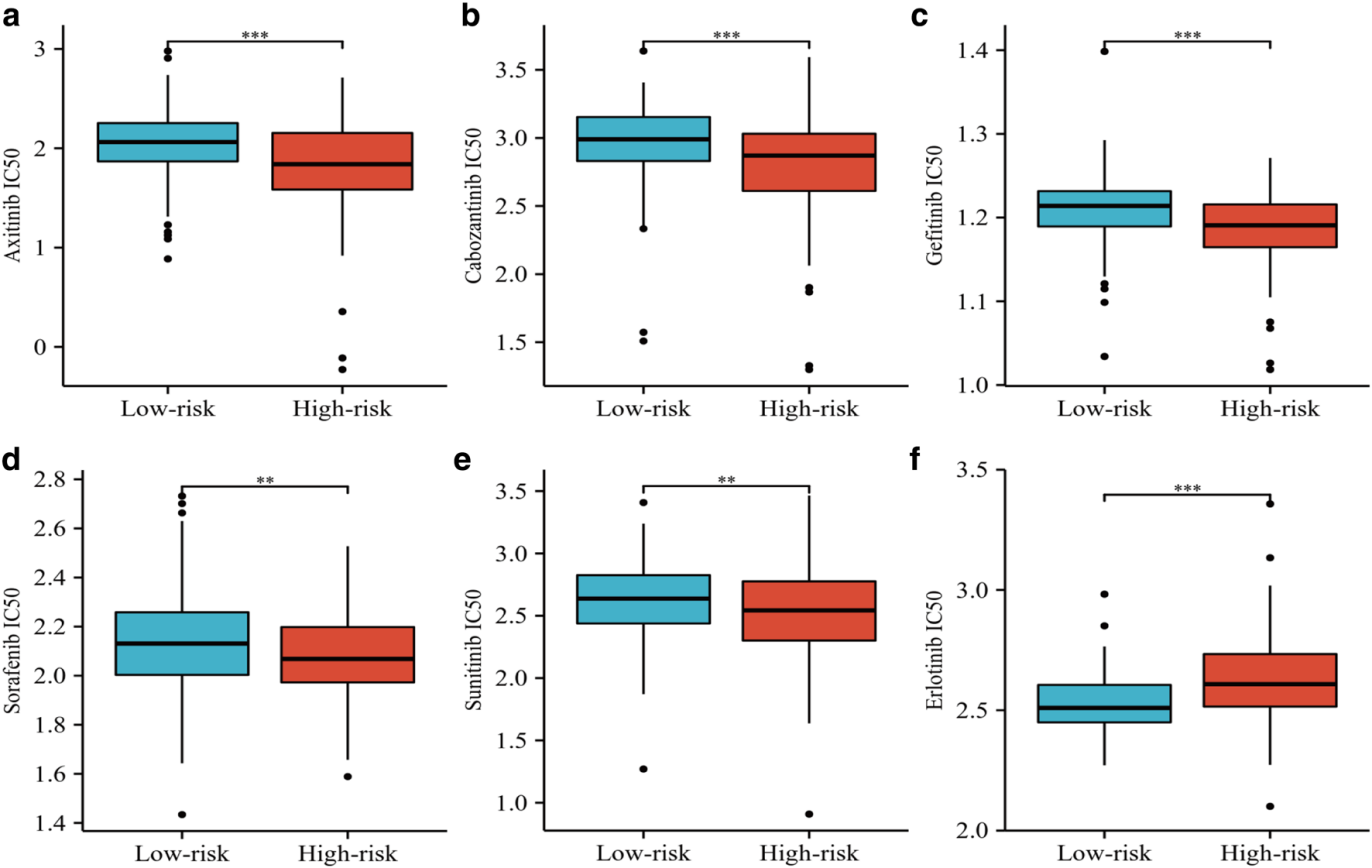
Therapeutic response of HCC patients to targeted drugs. (**a**) Axitinib. (**b**) Cabozantinib. (**c**) Gefitinib. (**d**) Sorafanib. (**e**) Sunitinib. (**f**) Erlotinib. (**P < 0.01, ***P < 0.001).

Axitinib (Fig. 7a), Cabozantinib (Fig. 7b), Gefitinib (Fig. 7c), Sorafanib (Fig. 7d), and Sunitinib (Fig. 7e) all had greater IC50 values in the low-risk group than in the high-risk group, but Erlotinib's IC50 was lower in the low-risk group (Fig. 7f).

### RNA and protein expression status of EMTRGs

**Figure 8:**
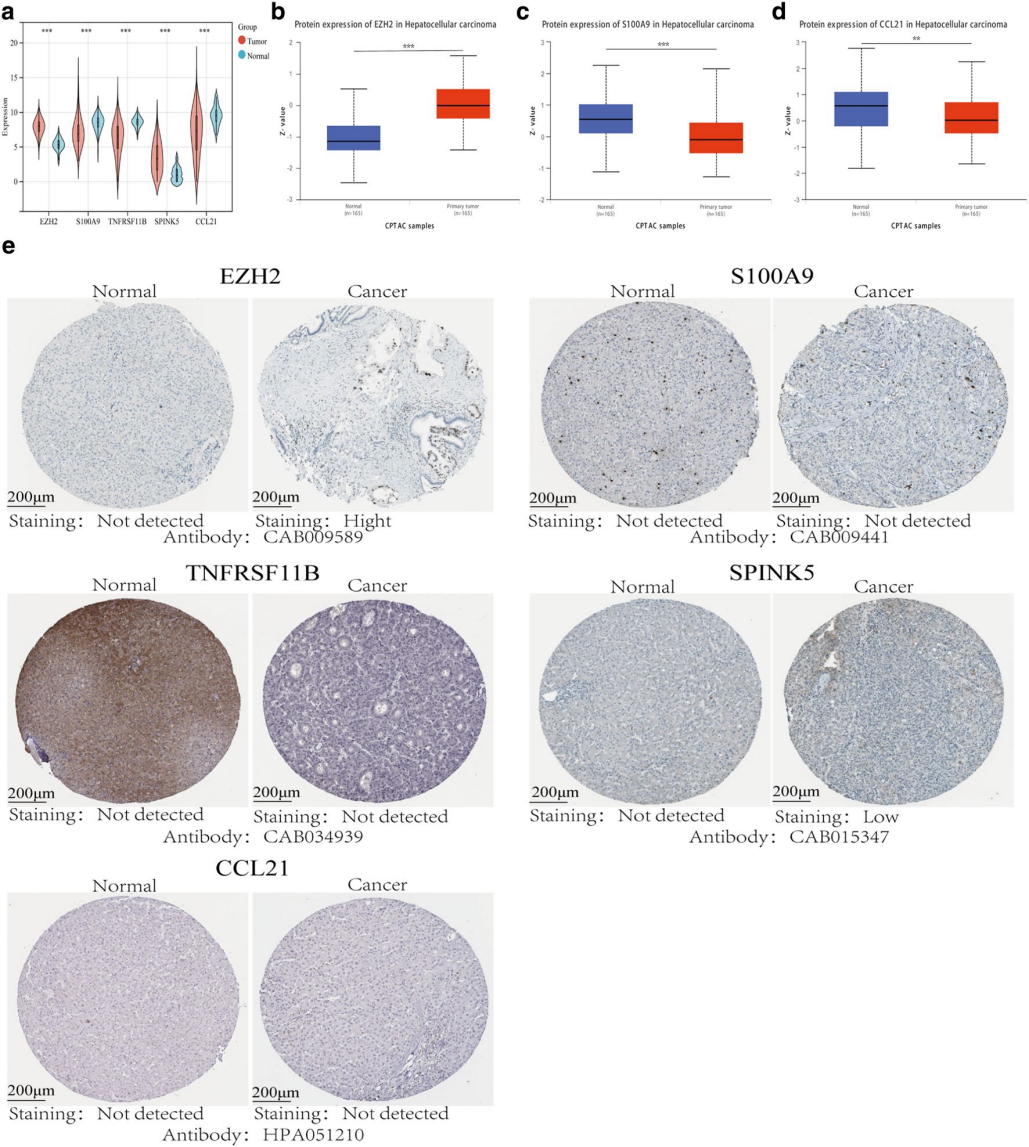
RNA and protein expression levels of EMTRGs. (**a**) RNA expression levels of EMTRGs in the TCGA cohort. (**b**) EZH2 protein expression levels in the CPTAC cohort. (**c**) S100A9 protein expression levels in the CPTAC cohort. (**d**) CCL21 protein expression levels in the CPTAC cohort. (**e**) Human protein atlas database (**P < 0.01, ***P < 0.001).

The expressions of S100A9, TNFRSF11B, and CCL21 was down-regulated in the five EMTRGs that the TCGA cohort was utilized to build the risk model, but EZH2 and SPINK5 expression was up-regulated (Fig. 8a). The expressions of S100A9 and CCL21 were downregulated while the expression of EZH2 were elevated at the protein level in the UALCAN database CPTAC cohort, with the exception of the absence of TNFRSF11B and SPINK5 data (Fig. 8b–d). Next, the expressions of S100A9, TNFRSF11B, CCL21, EZH2, and SPINK5 were further investigated using the Human Protein Atlas database with immunohistochemistry methods. The protein expression levels of EZH2 and SPINK5 were higher in HCC tissues compared with paracarcinoma tissues, and there was no significant difference in the protein expression levels of S100A9, TNFRSF11B and CCL21 in paracarcinoma and HCC tissues (Fig. 8e). Although the precise changes in EMTRGs between HCC and paracarcinoma tissues cannot yet be determined, the primary results indicate that S100A9 and CCL21 are down-regulated in HCC whereas EZH2 and SPINK5 are both up-regulated at the RNA and protein levels. Experimental confirmation of TNFRSF11B expression level is required.

## Disscussion

Liver cancer is currently a difficult diagnostic and therapeutic problem in the medical field, and has been the focus of research, such as high incidence and recurrence rates, poor prognosis, how to treat early and monitor recurrence. While liver transplants can cure liver cancer to some extent, resources are limited, and the vast majority of cases do not meet the conditions for liver transfers. In addition, due to the rapid proliferation and growth of tumor cells, different cell types and structural characteristics may appear within HCC lesions, such as different degrees of differentiation, glandular duct structure, cystic changes, and inflammatory cell infiltration. These morphological heterogeneities make the diagnosis and treatment of HCC more challenging. Therefore, it is essential to investigate ways to increase liver cancer early detection and forecast the therapeutic effectiveness and survival rates of patients. In recent years, an increasing number of studies have reported prognostic models for tumors. In this study, we had developed five EMTRGs predictive models based on the TCGA cohort, and ICGC cohort validation produced similar outcomes. Patients with high-risk HCC had a worse prognosis and a greater mortality rate than those with low-risk group. With increases in Phase T, Grade, clinical stage, and immunity scores, risk scores also increased significantly. Our results demonstrate that the risk scores of the five EMTRGs can be used as a reliable independent predictor for patients with HCC.

In fact, five EMTRGs in the risk score model have been shown to be related to EMT processes in multiple malignant tumors and play different roles in tumors. EZH2, as a group protein methyltransferase, could influence the chromosome structure through epigenetic modification of the group protein-methylation, inhibit downstream target gene expression^33^, regulate liver metabolism and liver fibrosis, and regulate the development of liver cell cancer^34^. A previously report showed that EZH2 expression was upregulated in HCC and that silencing EZH2 inhibited HCC viability, migration, and invasion, increased E-cadherin expression, and decreased N-cadherin and waveform protein expression^35^. High expression of EZH2 in prostate cancer has been shown to be a biomarker indicating poor prognosis^36,37^. This suggests that EZH2 may contribute to EMT, which also supports the accuracy of the predictive characteristics of 5 EMTRGs in HCC. S100A9 is a member of the S100 protein family of calcium-binding proteins^38^, which binds Ca^2+^, Zn^2+^, RAGE, TLR4, and MMPs highly selectively, plays a regulatory role both intracellularly and extracellularly^39^, and is able to participate in the processes of cellular differentiation, signal transduction, migration, and adhesion^40,41^. Previous studies have shown that S100A9 can be used as a biomarker for diagnosing tumors such as hepatocellular carcinoma^42^, colorectal cancer^43,44^, gastric cancer^45,46^, oral squamous cell cancer^47^, and neuropathycoma^48^. It was found that the expression of S100A9 was higher in HBV-positive HCC tissues than in HBV-negative tissues, and silencing S100A9 expression blocked HBx (HBV encoded X protein)-induced growth and metastasis of HepG2 cells in vitro and in vivo. Meanwhile, the expression level of serum S100A9 was correlated with the TNM stage, extrahepatic metastasis status, and HBV DNA load of HBV-related HCC, which has good diagnostic value for identifying extrahepatic metastasis^49^. S100A9 also promotes HCC growth and metastasis through RACE-mediated ERK1/2 and P58 MAPK pathways^50^. Elevated levels of S100A9 expression in serum imply a poor prognosis for patients after radical resection for HCC^51^. TNFRSF11B (a member of the tumor necrosis factor receptor superfamily), also known as osteoprotegerin (OPG), is able to bind to nuclear factor-κB receptor-activating factor ligand (RANKL), which plays a key role in bone remodeling, but new studies have demonstrated that TNFRSF11B's role in tumors extends far beyond the role of a bone-specific regulator^52^. Real-time PCR examination of tumor tissues and paracancerous tissues from 40 patients with liver cancer revealed that TNFRSF11B was highly expressed in liver cancer tissues^53^. A report found that HCC patients with high TNFRSF11B expression had a lower survival rate and that TNFRSF11B was an independent risk factor for HCC^54^. Patients with increased TNFRSF11B expression in malignancies such as oral squamous cell carcinoma and prostate cancer have a worse prognosis^52^. SPINK5 contains 15 potentially functionally repressive regions, and its encoded proteins play important roles in the morphogenesis of skin and hair and in the anti-inflammatory and anti-microbial invasion of mucosal epithelium^55,56^. On the other hand, SPINK5 acted as a tumor suppressor in esophageal cancer, and which could inhibit the proliferation, migration, and invasion of esophageal cancer cells by inhibiting the Wnt/β-catenin signaling pathway and may serve as a therapeutic target for esophageal cancer^57^. Suwei et al. found that the downregulation of SPINK5 expression in melanoma could decrease E-cadherin expression, increase mesenchymal markers, and promote the EMT process^58^. One of the ligands of the CCR7 receptor, CCL21 is a significant member of the CCL family of chemokines^59^. CCL21-CCR7 could promote lymph node metastasis in esophageal squamous cell carcinoma through upregulation of MUC1^60^, and also increases the expression of MMP-2 and MMP-9 to promote proliferation, migration, and invasion of bladder cancer cells^61^. In breast cancer, TGF-β1 could induce the expression of CCL21 in lymphatic vessel endothelial cells and promote the EMT process in a paracrine manner^62^. However, the specific mechanisms of SPINK5 and CCL21 involvement in the EMT process in HCC are still to be further studied in vivo and in vitro. The above studies suggest that five EMTRGs in the prediction model have potential as prognostic biomarkers for HCC. In our study, both RNA and protein levels of EZH2 and SPINK5 were up-regulated in HCC, both S100A9 and CCL21 were down-regulated, and the expression level of TNFRSF11B was not verified. The different expression status reflects the different transcriptional levels of risk model genes in HCC, and the specific mechanisms and roles of this genes need to be further investigated.

By GSEA analysis, it was found that the high-risk group was predominantly enriched for tumor-related pathway, such as the P53 signaling pathway, bladder cancer, and mismatch repair, suggesting that tumors in the high-risk group were more aggressive. This is consistent with the clinical characteristics of the high-risk group, such as hypofractionation, late pathological and clinical staging, poor prognosis, and high mortality. On the other hand, the pathways enriched in the low-risk group are mainly related to the physiological metabolism of the liver, such as fatty acid metabolism, primary bile acid biosynthesis, and histidine metabolism. The liver is the central organ for fatty acid metabolism, and its metabolic pathways mainly include β- oxidation, ω- oxidation and fatty acid synthesis, etc. The β- oxidation pathway of fatty acids is one of the important lipid metabolism pathways in organisms, and its regulation mechanism is complex and involves the participation of multiple enzymes. In addition, the liver is also the main site for primary bile acid biosynthesis and histidine metabolism. Therefore, a better clinical outcome was favored compared with the high-risk group. Additionally, this study revealed that when the risk score climbed, metabolism-related pathways declined while tumor-related pathways increased. According to this, risk scores may be able to forecast a patient's prognosis for HCC and help researchers better understand the molecular processes that lead to the formation of HCC.

The tumor microenvironment (TME) is the cellular environment in which a tumor exists, including the tumor itself, as well as surrounding blood vessels, extracellular matrix, surrounding normal cells, and associated signaling molecules, and is of great significance to tumor invasion and metastasis^63^. The TME's most prevalent population of tumor-infiltrating immune cells, tumor-associated macrophages (TAMs), is a crucial part of the TME^64^. TAMs were discovered to speed up the development of HCC by secreting a number of cytokines, inducing EMT, and enhancing the proliferation, invasion, and migration of tumor^65^. The IL-6 secreted by TAMs can promote tumor transfer by activating the downstream JAK/STAT3 signal pathway, downregulating E-caderin, and upregulating the expression of EMT-related transcription factors such as vimentin, snail, and twist^66^. In addition, TNF-α secreted by TAMs activates downstream the Wnt/β-catenin signal pathway, causing HCC to occur EMT^67^. In addition to secreting cell factors, TAMs also secrete exosomes and S100A9, thereby regulating the stem cell properties of tumor cells^68,69^. The results of this study suggest that the EMTRGs risk model is significantly associated with immune cell infiltration and immune subtypes, and hypothesize the difference in prognosis between the high-risk and low-risk groups may be due to the different immune statuses of the patients.

The high malignancy of HCC lies not only in its ability to metastasize but also in its insensitivity to chemotherapeutic agents. It was found that HCC patients with resistance to sorafenib are linked to the degree of liver cancer cell EMT^70,71^. miR-216a/217 induces EMT formation through regulation of TGF-β and PI3K/AKT signaling pathways, leading to resistance to sorafenib in HCC patients. Down-regulation of miR-216a/217 expression can block the activation of TGF-β pathway to overcome the resistance generated by EMT^72^. HGF stimulates the P-ERK/Snail/EMT and P-STAT3/ Snail/EMT pathways in HCC to induce resistance to sorafenib. However, regorafenib inhibits P-ERK and P-STAT3 to block the HGF-induced EMT process, which in turn inhibits HGF-induced resistance to sorafenib^73^. The high-risk group may be more responsive to treatment with Axitinib, Cabozantinib, Gefitinib, Sorafanib, and Sunitinib, according to the results of our drug sensitivity prediction research. In contrast, patients in the low-risk group might profit from Erlotinib therapy. Therefore, patients in the high-risk group may be better suited for targeted therapeutic agents, and patients in the low-risk group have limited treatment options.

Invasive metastasis is the most important biological feature of malignant tumors and is a key determinant of patient prognosis, and EMT has attracted widespread attention as a potential mechanism of tumor cell metastasis. The RNA and protein expression levels of these genes in HCC were initially investigated. however, this study is needed to be upgraded. Only two databases were used for the construction and validation of these five EMTRGs risk model, and cellular or animal experiments are needed to confirm the precise regulatory mechanisms of the prognostic features of EMTRGs. In the meantime, the results of this study are based on transcriptomic profiling, and the risk model developed for EMTRGs has not been clinically applied or generalized. The role of EMTRGs risk models in HCC is the focus of our subsequent studies.

## Conclusions

In conclusion, the TCGA database was used to build a novel risk model for EMTRGs, which was then verified in the ICGC database. The model can be used to predict the prognosis of HCC patients as a key factor in treatment. The results of this study provide deeper insights into the role of these key prognostic factors in HCC and provide some support for their future use as potential diagnostic and therapeutic biomarkers for HCC, laying some foundation for future clinical applications.

### Supplementary Information

Supplementary Information.

## Data Availability

The datasets generated and analyzed during the present study are available and are accessible from corresponding author.
