# Supplementary material for: EMT-related gene risk model establishment for prognosis and drug treatment efficiency prediction in hepatocellular carcinoma
Source: Sci Rep. 2023 Nov 21;13:20380. doi: 10.1038/s41598-023-47886-z (PMC10663558; doi:10.1038/s41598-023-47886-z)
Supplement: Supplementary file 1 — Supplementary Information. [file 41598_2023_47886_MOESM1_ESM.docx]

Supplementary data 1:

Sample ID:

| TCGA Cohort | ICGC Cohort |
| --- | --- |
| TCGA-2Y-A9GS | DO227643 |
| TCGA-2Y-A9GT | DO227801 |
| TCGA-2Y-A9GU | DO23508 |
| TCGA-2Y-A9GV | DO23509 |
| TCGA-2Y-A9GW | DO23510 |
| TCGA-2Y-A9GX | DO23511 |
| TCGA-2Y-A9GY | DO23512 |
| TCGA-2Y-A9GZ | DO23513 |
| TCGA-2Y-A9H0 | DO23514 |
| TCGA-2Y-A9H1 | DO23515 |
| TCGA-2Y-A9H2 | DO23516 |
| TCGA-2Y-A9H3 | DO23517 |
| TCGA-2Y-A9H4 | DO23518 |
| TCGA-2Y-A9H5 | DO23519 |
| TCGA-2Y-A9H6 | DO23521 |
| TCGA-2Y-A9H7 | DO23523 |
| TCGA-2Y-A9H8 | DO23524 |
| TCGA-2Y-A9H9 | DO23525 |
| TCGA-2Y-A9HA | DO23526 |
| TCGA-2Y-A9HB | DO23527 |
| TCGA-3K-AAZ8 | DO23528 |
| TCGA-4R-AA8I | DO23529 |
| TCGA-5C-A9VG | DO23530 |
| TCGA-5C-A9VH | DO23531 |
| TCGA-5C-AAPD | DO23532 |
| TCGA-5R-AA1C | DO23533 |
| TCGA-5R-AA1D | DO23534 |
| TCGA-5R-AAAM | DO23535 |
| TCGA-BC-4072 | DO23536 |
| TCGA-BC-4073 | DO23537 |
| TCGA-BC-A10Q | DO23538 |
| TCGA-BC-A10R | DO23539 |
| TCGA-BC-A10S | DO23540 |
| TCGA-BC-A10T | DO23541 |
| TCGA-BC-A10U | DO23542 |
| TCGA-BC-A10W | DO23543 |
| TCGA-BC-A10X | DO23544 |
| TCGA-BC-A10Y | DO23545 |
| TCGA-BC-A10Z | DO23546 |
| TCGA-BC-A110 | DO23547 |
| TCGA-BC-A112 | DO23548 |
| TCGA-BC-A216 | DO23549 |
| TCGA-BC-A217 | DO23550 |
| TCGA-BC-A3KF | DO23551 |
| TCGA-BC-A3KG | DO23552 |
| TCGA-BC-A5W4 | DO45091 |
| TCGA-BC-A69H | DO45092 |
| TCGA-BC-A69I | DO45093 |
| TCGA-BC-A8YO | DO45094 |
| TCGA-BD-A2L6 | DO45095 |
| TCGA-BD-A3EP | DO45096 |
| TCGA-BD-A3ER | DO45097 |
| TCGA-BW-A5NO | DO45099 |
| TCGA-CC-5258 | DO45101 |
| TCGA-CC-5259 | DO45103 |
| TCGA-CC-5260 | DO45105 |
| TCGA-CC-5261 | DO45107 |
| TCGA-CC-5262 | DO45109 |
| TCGA-CC-5263 | DO45111 |
| TCGA-CC-5264 | DO45113 |
| TCGA-CC-A123 | DO45115 |
| TCGA-CC-A1HT | DO45117 |
| TCGA-CC-A3M9 | DO45119 |
| TCGA-CC-A3MA | DO45121 |
| TCGA-CC-A3MB | DO45123 |
| TCGA-CC-A3MC | DO45125 |
| TCGA-CC-A5UC | DO45127 |
| TCGA-CC-A5UD | DO45129 |
| TCGA-CC-A5UE | DO45131 |
| TCGA-CC-A7IE | DO45133 |
| TCGA-CC-A7IF | DO45135 |
| TCGA-CC-A7IG | DO45137 |
| TCGA-CC-A7IH | DO45139 |
| TCGA-CC-A7II | DO45141 |
| TCGA-CC-A7IJ | DO45145 |
| TCGA-CC-A7IK | DO45149 |
| TCGA-CC-A7IL | DO45153 |
| TCGA-CC-A8HS | DO45155 |
| TCGA-CC-A8HT | DO45157 |
| TCGA-CC-A8HU | DO45159 |
| TCGA-CC-A8HV | DO45161 |
| TCGA-CC-A9FS | DO45163 |
| TCGA-CC-A9FW | DO45165 |
| TCGA-DD-A113 | DO45167 |
| TCGA-DD-A114 | DO45169 |
| TCGA-DD-A115 | DO45171 |
| TCGA-DD-A116 | DO45173 |
| TCGA-DD-A118 | DO45175 |
| TCGA-DD-A119 | DO45177 |
| TCGA-DD-A11A | DO45179 |
| TCGA-DD-A11B | DO45181 |
| TCGA-DD-A11C | DO45183 |
| TCGA-DD-A11D | DO45185 |
| TCGA-DD-A1EA | DO45187 |
| TCGA-DD-A1EB | DO45189 |
| TCGA-DD-A1EC | DO45191 |
| TCGA-DD-A1ED | DO45193 |
| TCGA-DD-A1EE | DO45195 |
| TCGA-DD-A1EF | DO45197 |
| TCGA-DD-A1EG | DO45199 |
| TCGA-DD-A1EH | DO45201 |
| TCGA-DD-A1EI | DO45203 |
| TCGA-DD-A1EJ | DO45205 |
| TCGA-DD-A1EK | DO45207 |
| TCGA-DD-A1EL | DO45209 |
| TCGA-DD-A39V | DO45211 |
| TCGA-DD-A39W | DO45213 |
| TCGA-DD-A39X | DO45215 |
| TCGA-DD-A39Y | DO45217 |
| TCGA-DD-A39Z | DO45219 |
| TCGA-DD-A3A1 | DO45221 |
| TCGA-DD-A3A2 | DO45223 |
| TCGA-DD-A3A3 | DO45225 |
| TCGA-DD-A3A4 | DO45227 |
| TCGA-DD-A3A5 | DO45229 |
| TCGA-DD-A3A6 | DO45231 |
| TCGA-DD-A3A7 | DO45233 |
| TCGA-DD-A3A8 | DO45235 |
| TCGA-DD-A3A9 | DO45237 |
| TCGA-DD-A4NA | DO45239 |
| TCGA-DD-A4NB | DO45241 |
| TCGA-DD-A4ND | DO45243 |
| TCGA-DD-A4NE | DO45245 |
| TCGA-DD-A4NF | DO45247 |
| TCGA-DD-A4NG | DO45249 |
| TCGA-DD-A4NH | DO45251 |
| TCGA-DD-A4NI | DO45253 |
| TCGA-DD-A4NJ | DO45255 |
| TCGA-DD-A4NK | DO45257 |
| TCGA-DD-A4NL | DO45259 |
| TCGA-DD-A4NN | DO45261 |
| TCGA-DD-A4NO | DO45263 |
| TCGA-DD-A4NP | DO45265 |
| TCGA-DD-A4NQ | DO45267 |
| TCGA-DD-A4NR | DO45269 |
| TCGA-DD-A4NS | DO45273 |
| TCGA-DD-A4NV | DO45275 |
| TCGA-DD-A73A | DO45277 |
| TCGA-DD-A73B | DO45279 |
| TCGA-DD-A73C | DO45281 |
| TCGA-DD-A73D | DO45283 |
| TCGA-DD-A73E | DO45285 |
| TCGA-DD-A73F | DO45287 |
| TCGA-DD-A73G | DO45289 |
| TCGA-DD-AA3A | DO45297 |
| TCGA-DD-AAC8 | DO45299 |
| TCGA-DD-AAC9 | DO45301 |
| TCGA-DD-AACA | DO45303 |
| TCGA-DD-AACB | DO45305 |
| TCGA-DD-AACC | DO45307 |
| TCGA-DD-AACD | DO48672 |
| TCGA-DD-AACE | DO48674 |
| TCGA-DD-AACF | DO48677 |
| TCGA-DD-AACG | DO48679 |
| TCGA-DD-AACH | DO48681 |
| TCGA-DD-AACI | DO48682 |
| TCGA-DD-AACJ | DO48693 |
| TCGA-DD-AACK | DO48695 |
| TCGA-DD-AACL | DO48697 |
| TCGA-DD-AACN | DO48700 |
| TCGA-DD-AACO | DO48701 |
| TCGA-DD-AACP | DO48704 |
| TCGA-DD-AACQ | DO48706 |
| TCGA-DD-AACS | DO48712 |
| TCGA-DD-AACT | DO48715 |
| TCGA-DD-AACU | DO48716 |
| TCGA-DD-AACV | DO48717 |
| TCGA-DD-AACW | DO48719 |
| TCGA-DD-AACX | DO48720 |
| TCGA-DD-AACY | DO48721 |
| TCGA-DD-AACZ | DO48723 |
| TCGA-DD-AAD0 | DO48725 |
| TCGA-DD-AAD1 | DO48727 |
| TCGA-DD-AAD2 | DO48728 |
| TCGA-DD-AAD3 | DO48730 |
| TCGA-DD-AAD5 | DO48732 |
| TCGA-DD-AAD6 | DO48733 |
| TCGA-DD-AAD8 | DO48736 |
| TCGA-DD-AADA | DO48737 |
| TCGA-DD-AADB | DO48741 |
| TCGA-DD-AADC | DO48742 |
| TCGA-DD-AADD | DO48743 |
| TCGA-DD-AADF | DO48746 |
| TCGA-DD-AADG | DO48747 |
| TCGA-DD-AADI | DO48751 |
| TCGA-DD-AADJ | DO48757 |
| TCGA-DD-AADK | DO48759 |
| TCGA-DD-AADL | DO48760 |
| TCGA-DD-AADM | DO48761 |
| TCGA-DD-AADN | DO50774 |
| TCGA-DD-AADO | DO50776 |
| TCGA-DD-AADP | DO50778 |
| TCGA-DD-AADQ | DO50783 |
| TCGA-DD-AADR | DO50785 |
| TCGA-DD-AADS | DO50787 |
| TCGA-DD-AADU | DO50789 |
| TCGA-DD-AADV | DO50791 |
| TCGA-DD-AADW | DO50793 |
| TCGA-DD-AADY | DO50798 |
| TCGA-DD-AAE0 | DO50799 |
| TCGA-DD-AAE1 | DO50800 |
| TCGA-DD-AAE2 | DO50802 |
| TCGA-DD-AAE3 | DO50803 |
| TCGA-DD-AAE4 | DO50804 |
| TCGA-DD-AAE6 | DO50805 |
| TCGA-DD-AAE7 | DO50806 |
| TCGA-DD-AAE9 | DO50807 |
| TCGA-DD-AAEA | DO50808 |
| TCGA-DD-AAEB | DO50809 |
| TCGA-DD-AAED | DO50811 |
| TCGA-DD-AAEE | DO50813 |
| TCGA-DD-AAEG | DO50814 |
| TCGA-DD-AAEH | DO50815 |
| TCGA-DD-AAEI | DO50816 |
| TCGA-DD-AAEK | DO50817 |
| TCGA-DD-AAVP | DO50818 |
| TCGA-DD-AAVQ | DO50819 |
| TCGA-DD-AAVR | DO50820 |
| TCGA-DD-AAVS | DO50822 |
| TCGA-DD-AAVU | DO50825 |
| TCGA-DD-AAVV | DO50829 |
| TCGA-DD-AAVW | DO50832 |
| TCGA-DD-AAVX | DO50839 |
| TCGA-DD-AAVY | DO50840 |
| TCGA-DD-AAVZ | DO50844 |
| TCGA-DD-AAW0 | DO50845 |
| TCGA-DD-AAW1 | DO50850 |
| TCGA-DD-AAW2 | DO50855 |
| TCGA-DD-AAW3 | DO50857 |
| TCGA-ED-A459 | DO50859 |
| TCGA-ED-A4XI |  |
| TCGA-ED-A5KG |  |
| TCGA-ED-A627 |  |
| TCGA-ED-A66X |  |
| TCGA-ED-A66Y |  |
| TCGA-ED-A7PX |  |
| TCGA-ED-A7PY |  |
| TCGA-ED-A7PZ |  |
| TCGA-ED-A7XO |  |
| TCGA-ED-A7XP |  |
| TCGA-ED-A82E |  |
| TCGA-ED-A8O5 |  |
| TCGA-ED-A8O6 |  |
| TCGA-ED-A97K |  |
| TCGA-EP-A12J |  |
| TCGA-EP-A26S |  |
| TCGA-EP-A2KA |  |
| TCGA-EP-A2KB |  |
| TCGA-EP-A2KC |  |
| TCGA-EP-A3JL |  |
| TCGA-EP-A3RK |  |
| TCGA-ES-A2HS |  |
| TCGA-ES-A2HT |  |
| TCGA-FV-A23B |  |
| TCGA-FV-A2QQ |  |
| TCGA-FV-A2QR |  |
| TCGA-FV-A3I0 |  |
| TCGA-FV-A3I1 |  |
| TCGA-FV-A3R2 |  |
| TCGA-FV-A3R3 |  |
| TCGA-FV-A495 |  |
| TCGA-FV-A496 |  |
| TCGA-FV-A4ZP |  |
| TCGA-FV-A4ZQ |  |
| TCGA-G3-A25S |  |
| TCGA-G3-A25T |  |
| TCGA-G3-A25U |  |
| TCGA-G3-A25V |  |
| TCGA-G3-A25X |  |
| TCGA-G3-A25Y |  |
| TCGA-G3-A25Z |  |
| TCGA-G3-A3CG |  |
| TCGA-G3-A3CH |  |
| TCGA-G3-A3CI |  |
| TCGA-G3-A3CJ |  |
| TCGA-G3-A3CK |  |
| TCGA-G3-A5SI |  |
| TCGA-G3-A5SJ |  |
| TCGA-G3-A5SK |  |
| TCGA-G3-A5SL |  |
| TCGA-G3-A5SM |  |
| TCGA-G3-A6UC |  |
| TCGA-G3-A7M5 |  |
| TCGA-G3-A7M6 |  |
| TCGA-G3-A7M7 |  |
| TCGA-G3-A7M8 |  |
| TCGA-G3-A7M9 |  |
| TCGA-G3-AAUZ |  |
| TCGA-G3-AAV0 |  |
| TCGA-G3-AAV1 |  |
| TCGA-G3-AAV2 |  |
| TCGA-G3-AAV3 |  |
| TCGA-G3-AAV4 |  |
| TCGA-G3-AAV5 |  |
| TCGA-G3-AAV6 |  |
| TCGA-G3-AAV7 |  |
| TCGA-GJ-A3OU |  |
| TCGA-GJ-A6C0 |  |
| TCGA-GJ-A9DB |  |
| TCGA-HP-A5MZ |  |
| TCGA-HP-A5N0 |  |
| TCGA-K7-A5RF |  |
| TCGA-K7-A5RG |  |
| TCGA-K7-A6G5 |  |
| TCGA-K7-AAU7 |  |
| TCGA-KR-A7K0 |  |
| TCGA-KR-A7K2 |  |
| TCGA-KR-A7K7 |  |
| TCGA-KR-A7K8 |  |
| TCGA-LG-A6GG |  |
| TCGA-LG-A9QC |  |
| TCGA-LG-A9QD |  |
| TCGA-MI-A75C |  |
| TCGA-MI-A75E |  |
| TCGA-MI-A75G |  |
| TCGA-MI-A75H |  |
| TCGA-MI-A75I |  |
| TCGA-MR-A520 |  |
| TCGA-MR-A8JO |  |
| TCGA-NI-A4U2 |  |
| TCGA-NI-A8LF |  |
| TCGA-O8-A75V |  |
| TCGA-PD-A5DF |  |
| TCGA-QA-A7B7 |  |
| TCGA-RC-A6M4 |  |
| TCGA-RC-A6M5 |  |
| TCGA-RC-A6M6 |  |
| TCGA-RC-A7S9 |  |
| TCGA-RC-A7SB |  |
| TCGA-RC-A7SF |  |
| TCGA-RC-A7SH |  |
| TCGA-RC-A7SK |  |
| TCGA-RG-A7D4 |  |
| TCGA-T1-A6J8 |  |
| TCGA-UB-A7MA |  |
| TCGA-UB-A7MB |  |
| TCGA-UB-A7MC |  |
| TCGA-UB-A7MD |  |
| TCGA-UB-A7ME |  |
| TCGA-UB-A7MF |  |
| TCGA-UB-AA0U |  |
| TCGA-UB-AA0V |  |
| TCGA-WJ-A86L |  |
| TCGA-WQ-A9G7 |  |
| TCGA-WQ-AB4B |  |
| TCGA-WX-AA44 |  |
| TCGA-WX-AA46 |  |
| TCGA-WX-AA47 |  |
| TCGA-XR-A8TC |  |
| TCGA-XR-A8TD |  |
| TCGA-XR-A8TE |  |
| TCGA-XR-A8TF |  |
| TCGA-XR-A8TG |  |
| TCGA-YA-A8S7 |  |
| TCGA-ZP-A9CV |  |
| TCGA-ZP-A9CY |  |
| TCGA-ZP-A9CZ |  |
| TCGA-ZP-A9D0 |  |
| TCGA-ZP-A9D1 |  |
| TCGA-ZP-A9D2 |  |
| TCGA-ZP-A9D4 |  |
| TCGA-ZS-A9CD |  |
| TCGA-ZS-A9CE |  |
| TCGA-ZS-A9CF |  |
| TCGA-ZS-A9CG |  |

Supplementary data 2:

EMT-related genes in paper:

| **1.EMT-related genes** | **2.different expression EMT-related genes** | **3.TCGA cohort Univariate Cox sigficant genes** | **4.TCGA cohort Lasso analysis sigficant genes** | **5.TCGA cohort Multivariate Cox sigficant genes** | |
| --- | --- | --- | --- | --- | --- |
| VIM | COL7A1 | MMP1 | MMP1 | **gene name** | **coefficient** |
| CDH1 | ITGA2 | SPP1 | SPP1 | EZH2 | 0.32088905 |
| FN1 | ITGA6 | EZH2 | EZH2 | S100A9 | 0.144247872 |
| ZEB1 | CYP27B1 | S100A9 | S100A9 | TNFRSF11B | 0.079363749 |
| CDH2 | H19 | ECM2 | PFN2 | SPINK5 | -0.096862316 |
| MMP2 | COL4A2 | PFN2 | FYN | CCL21 | -0.081919451 |
| SNAI2 | COL4A1 | FOXM1 | IGFBP4 |  |  |
| ZEB2 | LIPG | FYN | MAP7 |  |  |
| SPARC | ARTN | IGFBP4 | IGF1 |  |  |
| SNAI1 | DPT | C1S | ETS2 |  |  |
| CCN2 | IGFBP2 | METTL7A | BLNK |  |  |
| TWIST1 | IGFBP3 | MMP10 | TNFRSF11B |  |  |
| CDH11 | IGFBP4 | PRC1 | NQO1 |  |  |
| CLDN4 | KRTCAP3 | ESR1 | WNT5B |  |  |
| EPCAM | HAS2 | MAP7 | FAAH2 |  |  |
| SERPINE1 | TGFBR3 | STEAP4 | SPINK5 |  |  |
| TGFB1 | MRC2 | KRT17 | CCL21 |  |  |
| COL3A1 | LOX | IGF1 |  |  |  |
| ESRP1 | NTRK2 | MMP7 |  |  |  |
| INHBA | CAP2 | FBLN5 |  |  |  |
| PMP22 | CAPG | ETS2 |  |  |  |
| WNT5A | DBN1 | CTHRC1 |  |  |  |
| ST14 | DIO2 | TCF3 |  |  |  |
| TNC | CCL21 | GADD45B |  |  |  |
| CLDN7 | FGFR1 | MMP12 |  |  |  |
| EMP3 | NES | MATN3 |  |  |  |
| FSTL1 | PRSS22 | TRIM16 |  |  |  |
| ITGA5 | ZFPM2 | GJB3 |  |  |  |
| LAMC2 | MATN3 | BLNK |  |  |  |
| VCAN | NT5E | MCM7 |  |  |  |
| CDH3 | GNAL | SHMT1 |  |  |  |
| COL5A2 | SIX1 | CAPG |  |  |  |
| DCN | DACT1 | TNFRSF11B |  |  |  |
| DSP | EPB41L3 | IL1RN |  |  |  |
| ERBB3 | THBS1 | NQO1 |  |  |  |
| POSTN | C1S | ANO1 |  |  |  |
| RAB25 | SRGN | DLC1 |  |  |  |
| SPINT2 | PCOLCE | WNT5B |  |  |  |
| TGM2 | ZCCHC24 | CDKN2C |  |  |  |
| CDS1 | SAA1 | BCL9 |  |  |  |
| COL1A1 | ADAM15 | UCHL1 |  |  |  |
| ESRP2 | TNFRSF11B | RGS2 |  |  |  |
| GRHL2 | TMEM125 | ZCCHC24 |  |  |  |
| HTRA1 | SLC22A4 | GADD45A |  |  |  |
| MAP7 | AXL | AOX1 |  |  |  |
| MMP9 | IL13RA2 | TGFBR3 |  |  |  |
| OCLN | CD274 | TTC39A |  |  |  |
| PDGFRB | BCL9 | SYNE1 |  |  |  |
| PRSS8 | FAAH2 | CXCL1 |  |  |  |
| AP1M2 | STC1 | FERMT2 |  |  |  |
| AP1S2 | LAMA2 | EGF |  |  |  |
| AXL | WASF3 | TMEM30B |  |  |  |
| CD44 | F3 | EXPH5 |  |  |  |
| COL1A2 | STEAP4 | LRRC1 |  |  |  |
| COL5A1 | IFIT1 | HMGA2 |  |  |  |
| FBN1 | KRT17 | FAT4 |  |  |  |
| FGFBP1 | ACAA1 | ENAH |  |  |  |
| FGFR1 | LTBP4 | FAAH2 |  |  |  |
| FOXC2 | LTBP1 | SIX1 |  |  |  |
| IRF6 | CRLF1 | RHOB |  |  |  |
| KRT5 | PKP3 | LOX |  |  |  |
| PCOLCE | GLDC | PPL |  |  |  |
| S100A14 | L1CAM | SPINK5 |  |  |  |
| CLDN3 | PTPN3 | PAPSS2 |  |  |  |
| COL6A1 | FOSL1 | SOX5 |  |  |  |
| COL6A2 | FOXF2 | SOX2 |  |  |  |
| FXYD3 | TPD52L1 | HMOX1 |  |  |  |
| GNG11 | AOX1 | TET1 |  |  |  |
| GREM1 | PHGDH | PEA15 |  |  |  |
| IGFBP7 | PRRG4 | MMP9 |  |  |  |
| ITGB1 | EDIL3 | TP53I3 |  |  |  |
| KRT19 | RAB25 | PTHLH |  |  |  |
| LOX | NQO1 | ACAA1 |  |  |  |
| LOXL2 | FILIP1L | ANXA8 |  |  |  |
| PDGFRA | KRT19 | PTGS1 |  |  |  |
| PTX3 | PFN2 | CCL21 |  |  |  |
| SERPINE2 | TPM2 | ANGPTL4 |  |  |  |
| SLPI | TJP2 | CXCL6 |  |  |  |
| SPOCK1 | FYN |  |  |  |  |
| TCF4 | PTGS1 |  |  |  |  |
| TMEM30B | DCN |  |  |  |  |
| TPM1 | TRIM29 |  |  |  |  |
| TSPAN1 | FLRT2 |  |  |  |  |
| BMP1 | SLPI |  |  |  |  |
| C1ORF116 | ECM2 |  |  |  |  |
| CALD1 | ECM1 |  |  |  |  |
| CMTM3 | RGS2 |  |  |  |  |
| COL4A1 | BIK |  |  |  |  |
| CXCL1 | SNHG12 |  |  |  |  |
| CYP1B1 | GADD45A |  |  |  |  |
| DDR2 | FAT4 |  |  |  |  |
| DSC2 | SRPX |  |  |  |  |
| EHF | PEA15 |  |  |  |  |
| GLIPR1 | CKMT1A |  |  |  |  |
| HOOK1 | CKMT1B |  |  |  |  |
| IGFBP4 | CXCL12 |  |  |  |  |
| JUP | IL1RN |  |  |  |  |
| KRT18 | MME |  |  |  |  |
| LGALS1 | MACC1 |  |  |  |  |
| MAL2 | PTGER2 |  |  |  |  |
| MPZL2 | SCRIB |  |  |  |  |
| MST1R | LAMC2 |  |  |  |  |
| NR2F1 | FGFR2 |  |  |  |  |
| NRP1 | CXCL1 |  |  |  |  |
| PKP3 | CXCL6 |  |  |  |  |
| S100A8 | SCG2 |  |  |  |  |
| SEC31A | FERMT2 |  |  |  |  |
| SPINT1 | NEGR1 |  |  |  |  |
| TAGLN | KLC3 |  |  |  |  |
| THBS1 | PNMA2 |  |  |  |  |
| CCL2 | LEF1 |  |  |  |  |
| CD24 | CA2 |  |  |  |  |
| COL6A3 | FUT3 |  |  |  |  |
| CYBRD1 | FBLN5 |  |  |  |  |
| ECM1 | STRA6 |  |  |  |  |
| EGFR | NRG1 |  |  |  |  |
| F11R | SEMA5A |  |  |  |  |
| FAP | ETS2 |  |  |  |  |
| FBLN1 | MMP9 |  |  |  |  |
| FBLN5 | MMP7 |  |  |  |  |
| FERMT2 | LRRC1 |  |  |  |  |
| FGF2 | SHMT1 |  |  |  |  |
| GALNT3 | JUN |  |  |  |  |
| GAS1 | SCNN1A |  |  |  |  |
| GFPT2 | FAP |  |  |  |  |
| HAS2 | TMEM30B |  |  |  |  |
| HMGA2 | MMP1 |  |  |  |  |
| IGFBP3 | PRR16 |  |  |  |  |
| LSR | MXRA5 |  |  |  |  |
| LTBP1 | HMOX1 |  |  |  |  |
| MAP1B | EGF |  |  |  |  |
| MFF | ENAH |  |  |  |  |
| MMP1 | GADD45B |  |  |  |  |
| MMP3 | ESR1 |  |  |  |  |
| MYO5C | S100P |  |  |  |  |
| PLAT | MAP7 |  |  |  |  |
| PLAUR | TFPI2 |  |  |  |  |
| S100P | DLC1 |  |  |  |  |
| SRGN | ID4 |  |  |  |  |
| TGFBI | ID2 |  |  |  |  |
| TNFAIP6 | ID1 |  |  |  |  |
| TUBA1A | PI3 |  |  |  |  |
| ACKR3 | FOXM1 |  |  |  |  |
| COL4A2 | PTHLH |  |  |  |  |
| CRB3 | ESRP1 |  |  |  |  |
| CTNND1 | IGF2 |  |  |  |  |
| CXADR | IGF1 |  |  |  |  |
| DAB2 | SATB1 |  |  |  |  |
| DPYSL3 | SLC16A5 |  |  |  |  |
| DST | SFRP4 |  |  |  |  |
| ELF3 | EGR2 |  |  |  |  |
| EPB41L4B | NNMT |  |  |  |  |
| EPN3 | EXPH5 |  |  |  |  |
| F3 | SFRP1 |  |  |  |  |
| GADD45B | EPCAM |  |  |  |  |
| GRHL1 | KLK10 |  |  |  |  |
| IL1RN | BLNK |  |  |  |  |
| JAG1 | OSBPL3 |  |  |  |  |
| KRT14 | PVT1 |  |  |  |  |
| KRT15 | SPP1 |  |  |  |  |
| LAD1 | THBD |  |  |  |  |
| LAMA3 | HS3ST3B1 |  |  |  |  |
| LLGL2 | GJB3 |  |  |  |  |
| LTBP2 | CDKN2C |  |  |  |  |
| MAPK13 | CCL2 |  |  |  |  |
| MARVELD2 | HS3ST3A1 |  |  |  |  |
| MARVELD3 | PLEK2 |  |  |  |  |
| MISP | TP53I3 |  |  |  |  |
| MMP14 | IL1B |  |  |  |  |
| MRC2 | COMP |  |  |  |  |
| MUC1 | ADAM23 |  |  |  |  |
| MYL9 | RBMS3 |  |  |  |  |
| MYO5B | CBR3 |  |  |  |  |
| NT5C2 | ANXA3 |  |  |  |  |
| PMEPA1 | ANXA8 |  |  |  |  |
| PRRX1 | MCM7 |  |  |  |  |
| RGS4 | KRT7 |  |  |  |  |
| SCNN1A | P4HA2 |  |  |  |  |
| SFRP1 | THY1 |  |  |  |  |
| SH2D3A | ANGPTL4 |  |  |  |  |
| SH3YL1 | MMP10 |  |  |  |  |
| SRPX | MMP12 |  |  |  |  |
| TGFB1I1 | TCF3 |  |  |  |  |
| TGFB2 | GAS1 |  |  |  |  |
| THY1 | KLF10 |  |  |  |  |
| TMPRSS4 | PDGFRA |  |  |  |  |
| ABLIM1 | PROM2 |  |  |  |  |
| ACTA2 | PROM1 |  |  |  |  |
| ADAM12 | TET1 |  |  |  |  |
| AGR2 | BICC1 |  |  |  |  |
| AKT3 | GEM |  |  |  |  |
| ANGPTL2 | PDGFC |  |  |  |  |
| ANK3 | POU5F1 |  |  |  |  |
| ARHGAP8 | UCHL1 |  |  |  |  |
| BSPRY | WNT5B |  |  |  |  |
| CA2 | PAPSS2 |  |  |  |  |
| CAVIN1 | TRIM16 |  |  |  |  |
| CCN1 | TTC39A |  |  |  |  |
| CHN1 | SERPINE1 |  |  |  |  |
| CKMT1B | SOX10 |  |  |  |  |
| CLDN1 | EPPK1 |  |  |  |  |
| CLDN11 | PDLIM7 |  |  |  |  |
| COL7A1 | LAMA3 |  |  |  |  |
| CXCL12 | LAMA4 |  |  |  |  |
| DDR1 | PRC1 |  |  |  |  |
| DLC1 | POPDC3 |  |  |  |  |
| ELMO3 | METTL7A |  |  |  |  |
| EML1 | GALNT3 |  |  |  |  |
| EPB41L5 | ASCL2 |  |  |  |  |
| EPHA1 | LOXL2 |  |  |  |  |
| FA2H | XDH |  |  |  |  |
| FHL1 | PITPNM3 |  |  |  |  |
| ID2 | DOCK10 |  |  |  |  |
| IFIT3 | LY6E |  |  |  |  |
| IGFBP5 | CDH13 |  |  |  |  |
| ITGA6 | CDH12 |  |  |  |  |
| ITGB6 | SPINT2 |  |  |  |  |
| JAG2 | TACSTD2 |  |  |  |  |
| KLK10 | HMGA2 |  |  |  |  |
| LAMB3 | ZEB2 |  |  |  |  |
| LCN2 | TIAM1 |  |  |  |  |
| LEF1 | CTHRC1 |  |  |  |  |
| MLPH | NAP1L3 |  |  |  |  |
| MME | TMEM47 |  |  |  |  |
| MYH14 | AREG |  |  |  |  |
| NOTCH1 | WNT11 |  |  |  |  |
| NREP | ALDH1A3 |  |  |  |  |
| NT5E | SPINK5 |  |  |  |  |
| PALLD | SOX5 |  |  |  |  |
| PKP2 | OLFML3 |  |  |  |  |
| PLEK2 | RHOB |  |  |  |  |
| PLPP3 | S100A9 |  |  |  |  |
| PLS1 | S100A8 |  |  |  |  |
| PPL | TMC5 |  |  |  |  |
| PRR15L | ANO1 |  |  |  |  |
| PTHLH | WFDC2 |  |  |  |  |
| PXN | MCAM |  |  |  |  |
| QSOX1 | KLF4 |  |  |  |  |
| RECK | SNAI1 |  |  |  |  |
| SDC2 | SLC2A3 |  |  |  |  |
| SERPINB1 | CEACAM6 |  |  |  |  |
| STAP2 | SNCA |  |  |  |  |
| STC1 | LCN2 |  |  |  |  |
| TBX3 | SPOCK1 |  |  |  |  |
| TFPI2 | FGF2 |  |  |  |  |
| TIMP1 | SDC4 |  |  |  |  |
| TJP1 | CNKSR1 |  |  |  |  |
| TMC4 | HEY1 |  |  |  |  |
| TMC5 | PTGIS |  |  |  |  |
| UCHL1 | AKAP12 |  |  |  |  |
| VEGFC | GLS2 |  |  |  |  |
| WNT5B | JAG2 |  |  |  |  |
| ADAM15 | PRRX1 |  |  |  |  |
| ADAM19 | ANK3 |  |  |  |  |
| ADGRF1 | PPL |  |  |  |  |
| ADGRG1 | ADAMTS1 |  |  |  |  |
| AKAP12 | UCA1 |  |  |  |  |
| AKAP13 | LUM |  |  |  |  |
| ALPK2 | EZH2 |  |  |  |  |
| AOX1 | PRSS8 |  |  |  |  |
| ARHGAP17 | AQP3 |  |  |  |  |
| BICC1 | EPHB2 |  |  |  |  |
| BIK | SLC6A8 |  |  |  |  |
| BMP7 | FHL1 |  |  |  |  |
| BNC2 | CYBRD1 |  |  |  |  |
| C1S | CLDN11 |  |  |  |  |
| CD59 | NR2F1 |  |  |  |  |
| CELSR2 | SYNE1 |  |  |  |  |
| CFH | STEAP3 |  |  |  |  |
| CGN | GRHL2 |  |  |  |  |
| CKMT1A | TMPRSS13 |  |  |  |  |
| CTSZ | KCNMA1 |  |  |  |  |
| DACT1 | ABCA1 |  |  |  |  |
| DKK1 | DKK1 |  |  |  |  |
| DKK3 | HPS5 |  |  |  |  |
| DSG2 | SOX2 |  |  |  |  |
| EPPK1 | RGS4 |  |  |  |  |
| EVI2A | EPB41L4B |  |  |  |  |
| EXPH5 | BGN |  |  |  |  |
| FGFR2 | INHBA |  |  |  |  |
| FLNA | FAM110C |  |  |  |  |
| FLRT2 | MYLK |  |  |  |  |
| FST | ABCC4 |  |  |  |  |
| FSTL3 | SH3YL1 |  |  |  |  |
| FZD7 | PDGFA |  |  |  |  |
| GEM | KIAA0040 |  |  |  |  |
| GJB3 |  |  |  |  |  |
| GOLT1A |  |  |  |  |  |
| GRB7 |  |  |  |  |  |
| GSC |  |  |  |  |  |
| HS3ST3A1 |  |  |  |  |  |
| IFI44 |  |  |  |  |  |
| IFIT1 |  |  |  |  |  |
| IGFBP2 |  |  |  |  |  |
| IL4R |  |  |  |  |  |
| IRF7 |  |  |  |  |  |
| ITGAV |  |  |  |  |  |
| ITGB3 |  |  |  |  |  |
| JUN |  |  |  |  |  |
| KDF1 |  |  |  |  |  |
| KLK5 |  |  |  |  |  |
| KLK8 |  |  |  |  |  |
| KRT16 |  |  |  |  |  |
| KRT8 |  |  |  |  |  |
| LHFPL6 |  |  |  |  |  |
| LRRC1 |  |  |  |  |  |
| LUM |  |  |  |  |  |
| MAPKAP1 |  |  |  |  |  |
| MCAM |  |  |  |  |  |
| MRAS |  |  |  |  |  |
| MSRB3 |  |  |  |  |  |
| NECTIN4 |  |  |  |  |  |
| NEXN |  |  |  |  |  |
| NID1 |  |  |  |  |  |
| NID2 |  |  |  |  |  |
| OVOL2 |  |  |  |  |  |
| P3H1 |  |  |  |  |  |
| P3H2 |  |  |  |  |  |
| PDGFC |  |  |  |  |  |
| PERP |  |  |  |  |  |
| PLOD2 |  |  |  |  |  |
| PVR |  |  |  |  |  |
| RBM47 |  |  |  |  |  |
| RFTN1 |  |  |  |  |  |
| RHOD |  |  |  |  |  |
| S100A4 |  |  |  |  |  |
| SAA1 |  |  |  |  |  |
| SDC1 |  |  |  |  |  |
| SERPINB5 |  |  |  |  |  |
| SFRP4 |  |  |  |  |  |
| SHC1 |  |  |  |  |  |
| SLC2A3 |  |  |  |  |  |
| SLC6A8 |  |  |  |  |  |
| SLIT2 |  |  |  |  |  |
| SLIT3 |  |  |  |  |  |
| SMAD3 |  |  |  |  |  |
| SMPDL3B |  |  |  |  |  |
| SPRR1B |  |  |  |  |  |
| STAT1 |  |  |  |  |  |
| SULF1 |  |  |  |  |  |
| SYDE1 |  |  |  |  |  |
| SYK |  |  |  |  |  |
| TACSTD2 |  |  |  |  |  |
| TFPI |  |  |  |  |  |
| TGFB3 |  |  |  |  |  |
| THBS2 |  |  |  |  |  |
| TIMP2 |  |  |  |  |  |
| TIMP3 |  |  |  |  |  |
| TJP3 |  |  |  |  |  |
| TMEM125 |  |  |  |  |  |
| TMEM158 |  |  |  |  |  |
| TMPRSS11E |  |  |  |  |  |
| TPD52 |  |  |  |  |  |
| TPM2 |  |  |  |  |  |
| TRIM29 |  |  |  |  |  |
| VEGFA |  |  |  |  |  |
| WIPF1 |  |  |  |  |  |
| WWC1 |  |  |  |  |  |
| ZCCHC24 |  |  |  |  |  |
| ZFPM2 |  |  |  |  |  |
| ABCA12 |  |  |  |  |  |
| ABI1 |  |  |  |  |  |
| ACAA1 |  |  |  |  |  |
| ACTN1 |  |  |  |  |  |
| ADAM23 |  |  |  |  |  |
| ADAMTS1 |  |  |  |  |  |
| ADORA2B |  |  |  |  |  |
| ALDH1A3 |  |  |  |  |  |
| ALDH3A2 |  |  |  |  |  |
| ALOX5AP |  |  |  |  |  |
| ANGPTL4 |  |  |  |  |  |
| ANKRD22 |  |  |  |  |  |
| ANTXR1 |  |  |  |  |  |
| ANXA9 |  |  |  |  |  |
| APLP2 |  |  |  |  |  |
| AQP3 |  |  |  |  |  |
| AREG |  |  |  |  |  |
| ATP2C2 |  |  |  |  |  |
| ATP8B1 |  |  |  |  |  |
| B3GNT3 |  |  |  |  |  |
| BDNF |  |  |  |  |  |
| BGN |  |  |  |  |  |
| BIN1 |  |  |  |  |  |
| BPGM |  |  |  |  |  |
| C1ORF210 |  |  |  |  |  |
| CALB2 |  |  |  |  |  |
| CAP2 |  |  |  |  |  |
| CASK |  |  |  |  |  |
| CBLC |  |  |  |  |  |
| CCND2 |  |  |  |  |  |
| CD46 |  |  |  |  |  |
| CD68 |  |  |  |  |  |
| CDH12 |  |  |  |  |  |
| CDH13 |  |  |  |  |  |
| CDKN2C |  |  |  |  |  |
| CEACAM6 |  |  |  |  |  |
| CHST11 |  |  |  |  |  |
| COA1 |  |  |  |  |  |
| COL12A1 |  |  |  |  |  |
| COL13A1 |  |  |  |  |  |
| COL16A1 |  |  |  |  |  |
| COL17A1 |  |  |  |  |  |
| COL8A1 |  |  |  |  |  |
| CRLF1 |  |  |  |  |  |
| DENND2D |  |  |  |  |  |
| DSE |  |  |  |  |  |
| EDIL3 |  |  |  |  |  |
| EDN1 |  |  |  |  |  |
| EFEMP2 |  |  |  |  |  |
| ELF5 |  |  |  |  |  |
| ELK3 |  |  |  |  |  |
| ENAH |  |  |  |  |  |
| ENPP2 |  |  |  |  |  |
| EPHB2 |  |  |  |  |  |
| EPS8L1 |  |  |  |  |  |
| EPS8L2 |  |  |  |  |  |
| ERBB2 |  |  |  |  |  |
| EVPL |  |  |  |  |  |
| FBLN2 |  |  |  |  |  |
| FGFR3 |  |  |  |  |  |
| FMNL2 |  |  |  |  |  |
| FOXA2 |  |  |  |  |  |
| FOXC1 |  |  |  |  |  |
| GADD45A |  |  |  |  |  |
| GALNT10 |  |  |  |  |  |
| GJA1 |  |  |  |  |  |
| GNB4 |  |  |  |  |  |
| GOLGA2 |  |  |  |  |  |
| GPC6 |  |  |  |  |  |
| GRTP1 |  |  |  |  |  |
| HEG1 |  |  |  |  |  |
| HMOX1 |  |  |  |  |  |
| HRH1 |  |  |  |  |  |
| ID4 |  |  |  |  |  |
| IFI30 |  |  |  |  |  |
| IFITM3 |  |  |  |  |  |
| IGF1 |  |  |  |  |  |
| IGF1R |  |  |  |  |  |
| IL18 |  |  |  |  |  |
| INPP4B |  |  |  |  |  |
| ISG15 |  |  |  |  |  |
| ITGA2 |  |  |  |  |  |
| ITGA3 |  |  |  |  |  |
| ITGB4 |  |  |  |  |  |
| ITGB5 |  |  |  |  |  |
| JAM3 |  |  |  |  |  |
| KCNK1 |  |  |  |  |  |
| KIAA1191 |  |  |  |  |  |
| KIT |  |  |  |  |  |
| KRT17 |  |  |  |  |  |
| KRT7 |  |  |  |  |  |
| KRTCAP3 |  |  |  |  |  |
| LAMA2 |  |  |  |  |  |
| LAMA5 |  |  |  |  |  |
| LAMB1 |  |  |  |  |  |
| LARP6 |  |  |  |  |  |
| LAS1L |  |  |  |  |  |
| LIX1L |  |  |  |  |  |
| LMCD1 |  |  |  |  |  |
| LOXL1 |  |  |  |  |  |
| LRATD2 |  |  |  |  |  |
| LRP1 |  |  |  |  |  |
| LRRC15 |  |  |  |  |  |
| LTBP4 |  |  |  |  |  |
| LY6E |  |  |  |  |  |
| MACF1 |  |  |  |  |  |
| MAF |  |  |  |  |  |
| MAP4K4 |  |  |  |  |  |
| MARK3 |  |  |  |  |  |
| MBNL1 |  |  |  |  |  |
| MGP |  |  |  |  |  |
| MMP10 |  |  |  |  |  |
| MMP7 |  |  |  |  |  |
| MPP7 |  |  |  |  |  |
| MYLK |  |  |  |  |  |
| MYO6 |  |  |  |  |  |
| MYO9A |  |  |  |  |  |
| NAP1L3 |  |  |  |  |  |
| NDRG1 |  |  |  |  |  |
| NNMT |  |  |  |  |  |
| NPHP3 |  |  |  |  |  |
| NRG1 |  |  |  |  |  |
| NRP2 |  |  |  |  |  |
| OSBPL3 |  |  |  |  |  |
| PAK6 |  |  |  |  |  |
| PARD6B |  |  |  |  |  |
| PATJ |  |  |  |  |  |
| PEA15 |  |  |  |  |  |
| PIK3CD |  |  |  |  |  |
| PLAU |  |  |  |  |  |
| PROM2 |  |  |  |  |  |
| PRR16 |  |  |  |  |  |
| PTGIS |  |  |  |  |  |
| PTK2 |  |  |  |  |  |
| PTPN3 |  |  |  |  |  |
| PXDC1 |  |  |  |  |  |
| RAB31 |  |  |  |  |  |
| RAB6A |  |  |  |  |  |
| RASEF |  |  |  |  |  |
| RFLNB |  |  |  |  |  |
| SACS |  |  |  |  |  |
| SAT1 |  |  |  |  |  |
| SCEL |  |  |  |  |  |
| SCG2 |  |  |  |  |  |
| SDC4 |  |  |  |  |  |
| SERPINB2 |  |  |  |  |  |
| SHMT1 |  |  |  |  |  |
| SMAD2 |  |  |  |  |  |
| SMAD7 |  |  |  |  |  |
| SPHK1 |  |  |  |  |  |
| SPP1 |  |  |  |  |  |
| SPRR1A |  |  |  |  |  |
| SPRY1 |  |  |  |  |  |
| STEAP3 |  |  |  |  |  |
| SYNE2 |  |  |  |  |  |
| TCF3 |  |  |  |  |  |
| TGFBR2 |  |  |  |  |  |
| TGFBR3 |  |  |  |  |  |
| TIA1 |  |  |  |  |  |
| TJP2 |  |  |  |  |  |
| TMEM45B |  |  |  |  |  |
| TNFRSF12A |  |  |  |  |  |
| TOX3 |  |  |  |  |  |
| TP53I3 |  |  |  |  |  |
| TP63 |  |  |  |  |  |
| TPD52L1 |  |  |  |  |  |
| TPM4 |  |  |  |  |  |
| TTC28 |  |  |  |  |  |
| TTC39A |  |  |  |  |  |
| TWIST2 |  |  |  |  |  |
| UAP1 |  |  |  |  |  |
| UPP1 |  |  |  |  |  |
| VCAM1 |  |  |  |  |  |
| ZNF165 |  |  |  |  |  |
| ABCA1 |  |  |  |  |  |
| ABCB8 |  |  |  |  |  |
| ABCC4 |  |  |  |  |  |
| ACTA1 |  |  |  |  |  |
| ADIRF |  |  |  |  |  |
| AHNAK |  |  |  |  |  |
| AK5 |  |  |  |  |  |
| AKT1 |  |  |  |  |  |
| ALDH1A1 |  |  |  |  |  |
| ANKHD1 |  |  |  |  |  |
| ANKLE2 |  |  |  |  |  |
| ANKRD1 |  |  |  |  |  |
| ANO1 |  |  |  |  |  |
| ANPEP |  |  |  |  |  |
| ANXA3 |  |  |  |  |  |
| ANXA6 |  |  |  |  |  |
| ANXA8 |  |  |  |  |  |
| ARFGAP1 |  |  |  |  |  |
| ARHGEF40 |  |  |  |  |  |
| ARMCX1 |  |  |  |  |  |
| ARTN |  |  |  |  |  |
| B2M |  |  |  |  |  |
| B4GALT4 |  |  |  |  |  |
| BCL2A1 |  |  |  |  |  |
| BCL3 |  |  |  |  |  |
| BICDL2 |  |  |  |  |  |
| BIRC3 |  |  |  |  |  |
| BLNK |  |  |  |  |  |
| BMERB1 |  |  |  |  |  |
| BMP2 |  |  |  |  |  |
| CALU |  |  |  |  |  |
| CAPG |  |  |  |  |  |
| CAV1 |  |  |  |  |  |
| CAVIN2 |  |  |  |  |  |
| CBR3 |  |  |  |  |  |
| CCDC50 |  |  |  |  |  |
| CCND1 |  |  |  |  |  |
| CD9 |  |  |  |  |  |
| CD99L2 |  |  |  |  |  |
| CDH6 |  |  |  |  |  |
| CDK14 |  |  |  |  |  |
| CDKN1A |  |  |  |  |  |
| CEACAM1 |  |  |  |  |  |
| CEACAM5 |  |  |  |  |  |
| CNKSR1 |  |  |  |  |  |
| COBL |  |  |  |  |  |
| COLGALT2 |  |  |  |  |  |
| COMP |  |  |  |  |  |
| CORO1A |  |  |  |  |  |
| CPA4 |  |  |  |  |  |
| CSF1 |  |  |  |  |  |
| CTHRC1 |  |  |  |  |  |
| CXCL6 |  |  |  |  |  |
| CXCL8 |  |  |  |  |  |
| CXCR4 |  |  |  |  |  |
| CYP27B1 |  |  |  |  |  |
| CYP2F1 |  |  |  |  |  |
| CYP4F11 |  |  |  |  |  |
| DAPP1 |  |  |  |  |  |
| DBN1 |  |  |  |  |  |
| DEPTOR |  |  |  |  |  |
| DHRS2 |  |  |  |  |  |
| DIO2 |  |  |  |  |  |
| DIXDC1 |  |  |  |  |  |
| DMKN |  |  |  |  |  |
| DNAJB4 |  |  |  |  |  |
| DOCK10 |  |  |  |  |  |
| DOCK9 |  |  |  |  |  |
| DPP8 |  |  |  |  |  |
| DPT |  |  |  |  |  |
| ECM2 |  |  |  |  |  |
| EFCAB14 |  |  |  |  |  |
| EFHD2 |  |  |  |  |  |
| EFNA1 |  |  |  |  |  |
| EGF |  |  |  |  |  |
| EGR2 |  |  |  |  |  |
| EIF4A2 |  |  |  |  |  |
| EPDR1 |  |  |  |  |  |
| ERMP1 |  |  |  |  |  |
| ESR1 |  |  |  |  |  |
| ETS2 |  |  |  |  |  |
| FAAH2 |  |  |  |  |  |
| FAM110C |  |  |  |  |  |
| FAM83A |  |  |  |  |  |
| FAT4 |  |  |  |  |  |
| FBN2 |  |  |  |  |  |
| FGF5 |  |  |  |  |  |
| FILIP1L |  |  |  |  |  |
| FLNB |  |  |  |  |  |
| FUCA1 |  |  |  |  |  |
| FUT1 |  |  |  |  |  |
| FUT3 |  |  |  |  |  |
| FYN |  |  |  |  |  |
| GALE |  |  |  |  |  |
| GALNT2 |  |  |  |  |  |
| GGCT |  |  |  |  |  |
| GLDC |  |  |  |  |  |
| GLI3 |  |  |  |  |  |
| GLIPR2 |  |  |  |  |  |
| GLS2 |  |  |  |  |  |
| GLYR1 |  |  |  |  |  |
| GNAL |  |  |  |  |  |
| GPC1 |  |  |  |  |  |
| GSDME |  |  |  |  |  |
| GSE1 |  |  |  |  |  |
| GSK3B |  |  |  |  |  |
| HACL1 |  |  |  |  |  |
| HBEGF |  |  |  |  |  |
| HEY1 |  |  |  |  |  |
| HMBS |  |  |  |  |  |
| HPS5 |  |  |  |  |  |
| HS3ST1 |  |  |  |  |  |
| HS3ST3B1 |  |  |  |  |  |
| HSPG2 |  |  |  |  |  |
| IER3 |  |  |  |  |  |
| IFIT2 |  |  |  |  |  |
| IGF2 |  |  |  |  |  |
| IKBIP |  |  |  |  |  |
| IL11 |  |  |  |  |  |
| IL13RA2 |  |  |  |  |  |
| IL1B |  |  |  |  |  |
| IL1R1 |  |  |  |  |  |
| IL32 |  |  |  |  |  |
| ILDR1 |  |  |  |  |  |
| ILK |  |  |  |  |  |
| INAVA |  |  |  |  |  |
| INHBB |  |  |  |  |  |
| IQCB1 |  |  |  |  |  |
| ITGA11 |  |  |  |  |  |
| KCNMA1 |  |  |  |  |  |
| KDELR3 |  |  |  |  |  |
| KIAA0040 |  |  |  |  |  |
| KLC3 |  |  |  |  |  |
| KLF10 |  |  |  |  |  |
| KLK6 |  |  |  |  |  |
| KLK7 |  |  |  |  |  |
| KRT6B |  |  |  |  |  |
| LAMA4 |  |  |  |  |  |
| LBH |  |  |  |  |  |
| LCP1 |  |  |  |  |  |
| LIPG |  |  |  |  |  |
| LRATD1 |  |  |  |  |  |
| MAFB |  |  |  |  |  |
| MANSC1 |  |  |  |  |  |
| MAP3K7 |  |  |  |  |  |
| MATN2 |  |  |  |  |  |
| MATN3 |  |  |  |  |  |
| MCM7 |  |  |  |  |  |
| MEST |  |  |  |  |  |
| METTL7A |  |  |  |  |  |
| MFAP5 |  |  |  |  |  |
| MITF |  |  |  |  |  |
| MMP12 |  |  |  |  |  |
| MMP13 |  |  |  |  |  |
| MPRIP |  |  |  |  |  |
| MSN |  |  |  |  |  |
| MUC20 |  |  |  |  |  |
| MXRA5 |  |  |  |  |  |
| NAV1 |  |  |  |  |  |
| NDN |  |  |  |  |  |
| NECTIN3 |  |  |  |  |  |
| NEGR1 |  |  |  |  |  |
| NFATC1 |  |  |  |  |  |
| NQO1 |  |  |  |  |  |
| NRIP3 |  |  |  |  |  |
| NUAK1 |  |  |  |  |  |
| OGDH |  |  |  |  |  |
| OLFML3 |  |  |  |  |  |
| OVOL1 |  |  |  |  |  |
| P4HA2 |  |  |  |  |  |
| P4HA3 |  |  |  |  |  |
| PAFAH1B1 |  |  |  |  |  |
| PAG1 |  |  |  |  |  |
| PAM |  |  |  |  |  |
| PAPSS2 |  |  |  |  |  |
| PDGFA |  |  |  |  |  |
| PDLIM7 |  |  |  |  |  |
| PHGDH |  |  |  |  |  |
| PI3 |  |  |  |  |  |
| PKP1 |  |  |  |  |  |
| PLA2G7 |  |  |  |  |  |
| PLAAT4 |  |  |  |  |  |
| PLAGL1 |  |  |  |  |  |
| PLEKHO1 |  |  |  |  |  |
| PLPP2 |  |  |  |  |  |
| PNMA2 |  |  |  |  |  |
| POPDC3 |  |  |  |  |  |
| PPARG |  |  |  |  |  |
| PPFIBP2 |  |  |  |  |  |
| PPIB |  |  |  |  |  |
| PPP1R14C |  |  |  |  |  |
| PRC1 |  |  |  |  |  |
| PRKCA |  |  |  |  |  |
| PRKCH |  |  |  |  |  |
| PRKD1 |  |  |  |  |  |
| PROCR |  |  |  |  |  |
| PRR15 |  |  |  |  |  |
| PRR5-ARHGAP8 |  |  |  |  |  |
| PRRG4 |  |  |  |  |  |
| PRSS22 |  |  |  |  |  |
| PTAFR |  |  |  |  |  |
| PTGER2 |  |  |  |  |  |
| PTGS1 |  |  |  |  |  |
| RAB26 |  |  |  |  |  |
| RAPGEF5 |  |  |  |  |  |
| RBMS1 |  |  |  |  |  |
| RBMS3 |  |  |  |  |  |
| RGL1 |  |  |  |  |  |
| RGS2 |  |  |  |  |  |
| RHOB |  |  |  |  |  |
| RLN2 |  |  |  |  |  |
| RNF128 |  |  |  |  |  |
| RNF14 |  |  |  |  |  |
| ROR1 |  |  |  |  |  |
| RUNX2 |  |  |  |  |  |
| S100A9 |  |  |  |  |  |
| SBNO1 |  |  |  |  |  |
| SCRIB |  |  |  |  |  |
| SEC23A |  |  |  |  |  |
| SELENBP1 |  |  |  |  |  |
| SEMA3C |  |  |  |  |  |
| SEMA5A |  |  |  |  |  |
| SEPTIN6 |  |  |  |  |  |
| SERPINH1 |  |  |  |  |  |
| SFRP2 |  |  |  |  |  |
| SHROOM3 |  |  |  |  |  |
| SKIL |  |  |  |  |  |
| SLC16A5 |  |  |  |  |  |
| SLC22A4 |  |  |  |  |  |
| SLC37A2 |  |  |  |  |  |
| SLC3A2 |  |  |  |  |  |
| SLC7A5 |  |  |  |  |  |
| SMC5 |  |  |  |  |  |
| SNCA |  |  |  |  |  |
| SNTB1 |  |  |  |  |  |
| SOX10 |  |  |  |  |  |
| SOX2 |  |  |  |  |  |
| SPINK5 |  |  |  |  |  |
| ST3GAL2 |  |  |  |  |  |
| STAT5A |  |  |  |  |  |
| STEAP1 |  |  |  |  |  |
| STEAP4 |  |  |  |  |  |
| STRA6 |  |  |  |  |  |
| STX19 |  |  |  |  |  |
| STX3 |  |  |  |  |  |
| STXBP5 |  |  |  |  |  |
| SUSD5 |  |  |  |  |  |
| SYNE1 |  |  |  |  |  |
| SYT11 |  |  |  |  |  |
| SYTL1 |  |  |  |  |  |
| TBC1D23 |  |  |  |  |  |
| TC2N |  |  |  |  |  |
| TGFBR1 |  |  |  |  |  |
| THBD |  |  |  |  |  |
| TMEM126B |  |  |  |  |  |
| TMEM167A |  |  |  |  |  |
| TMEM37 |  |  |  |  |  |
| TMEM47 |  |  |  |  |  |
| TMPRSS13 |  |  |  |  |  |
| TNFAIP3 |  |  |  |  |  |
| TNFRSF11B |  |  |  |  |  |
| TOB1 |  |  |  |  |  |
| TSPAN13 |  |  |  |  |  |
| TSPAN4 |  |  |  |  |  |
| TUBA4A |  |  |  |  |  |
| TUFT1 |  |  |  |  |  |
| TUT7 |  |  |  |  |  |
| ULBP2 |  |  |  |  |  |
| VAV3 |  |  |  |  |  |
| VPS13A |  |  |  |  |  |
| VSNL1 |  |  |  |  |  |
| WFDC2 |  |  |  |  |  |
| WNK1 |  |  |  |  |  |
| WNT11 |  |  |  |  |  |
| XDH |  |  |  |  |  |
| XYLT1 |  |  |  |  |  |
| YAP1 |  |  |  |  |  |
| ZBTB38 |  |  |  |  |  |
| ZNF207 |  |  |  |  |  |
| ZNF788P |  |  |  |  |  |
| CTNNB1 |  |  |  |  |  |
| BMI1 |  |  |  |  |  |
| STAT3 |  |  |  |  |  |
| GLI1 |  |  |  |  |  |
| HIF1A |  |  |  |  |  |
| FOXQ1 |  |  |  |  |  |
| FOXM1 |  |  |  |  |  |
| MTDH |  |  |  |  |  |
| TBXT |  |  |  |  |  |
| SOX4 |  |  |  |  |  |
| SIX1 |  |  |  |  |  |
| NANOG |  |  |  |  |  |
| FAM3C |  |  |  |  |  |
| KLF17 |  |  |  |  |  |
| DCLK1 |  |  |  |  |  |
| TM4SF5 |  |  |  |  |  |
| KLF8 |  |  |  |  |  |
| WWTR1 |  |  |  |  |  |
| PDGFD |  |  |  |  |  |
| TDGF1 |  |  |  |  |  |
| EZH2 |  |  |  |  |  |
| CCR7 |  |  |  |  |  |
| SMAD4 |  |  |  |  |  |
| KDM1A |  |  |  |  |  |
| KLF4 |  |  |  |  |  |
| SLC39A6 |  |  |  |  |  |
| PTEN |  |  |  |  |  |
| CTNNBIP1 |  |  |  |  |  |
| EIF5A2 |  |  |  |  |  |
| BTBD7 |  |  |  |  |  |
| YBX1 |  |  |  |  |  |
| CCL18 |  |  |  |  |  |
| CTBP2 |  |  |  |  |  |
| CTBP1 |  |  |  |  |  |
| MACC1 |  |  |  |  |  |
| L1CAM |  |  |  |  |  |
| PROM1 |  |  |  |  |  |
| CUL4A |  |  |  |  |  |
| PARD3 |  |  |  |  |  |
| NEDD9 |  |  |  |  |  |
| SALL4 |  |  |  |  |  |
| SIRT1 |  |  |  |  |  |
| HDGF |  |  |  |  |  |
| PAQR3 |  |  |  |  |  |
| TET1 |  |  |  |  |  |
| SHH |  |  |  |  |  |
| PEBP1 |  |  |  |  |  |
| POU5F1 |  |  |  |  |  |
| SCUBE3 |  |  |  |  |  |
| FOSL1 |  |  |  |  |  |
| PCBP1 |  |  |  |  |  |
| MRTFA |  |  |  |  |  |
| TNFAIP8L2 |  |  |  |  |  |
| MAPK7 |  |  |  |  |  |
| FBXW7 |  |  |  |  |  |
| MTA1 |  |  |  |  |  |
| CCN5 |  |  |  |  |  |
| TRIM62 |  |  |  |  |  |
| SASH1 |  |  |  |  |  |
| PFN2 |  |  |  |  |  |
| DAB2IP |  |  |  |  |  |
| TRPS1 |  |  |  |  |  |
| MET |  |  |  |  |  |
| BTRC |  |  |  |  |  |
| EPB41L3 |  |  |  |  |  |
| WASF3 |  |  |  |  |  |
| OSM |  |  |  |  |  |
| SEMA4C |  |  |  |  |  |
| TIAM1 |  |  |  |  |  |
| EPAS1 |  |  |  |  |  |
| PDPN |  |  |  |  |  |
| SNAI3 |  |  |  |  |  |
| SATB1 |  |  |  |  |  |
| WNT1 |  |  |  |  |  |
| SOX9 |  |  |  |  |  |
| HTATIP2 |  |  |  |  |  |
| ASCL2 |  |  |  |  |  |
| FOXF2 |  |  |  |  |  |
| PARD6A |  |  |  |  |  |
| SOX5 |  |  |  |  |  |
| TRIM44 |  |  |  |  |  |
| TRIM16 |  |  |  |  |  |
| FOXD3 |  |  |  |  |  |
| DLX2 |  |  |  |  |  |
| PDCD4 |  |  |  |  |  |
| NTRK2 |  |  |  |  |  |
| RASAL2 |  |  |  |  |  |
| FZD2 |  |  |  |  |  |
| CIP2A |  |  |  |  |  |
| ID1 |  |  |  |  |  |
| BMP4 |  |  |  |  |  |
| FOXA1 |  |  |  |  |  |
| GKN1 |  |  |  |  |  |
| HCFC1R1 |  |  |  |  |  |
| USP22 |  |  |  |  |  |
| EFEMP1 |  |  |  |  |  |
| DYRK2 |  |  |  |  |  |
| PITPNM3 |  |  |  |  |  |
| ONECUT2 |  |  |  |  |  |
| TRIM66 |  |  |  |  |  |
| SUZ12 |  |  |  |  |  |
| SATB2 |  |  |  |  |  |
| CCL21 |  |  |  |  |  |
| JARID2 |  |  |  |  |  |
| TP53INP1 |  |  |  |  |  |
| BCL9 |  |  |  |  |  |
| GFER |  |  |  |  |  |
| RHOC |  |  |  |  |  |
| NODAL |  |  |  |  |  |
| FHOD1 |  |  |  |  |  |
| GOLM1 |  |  |  |  |  |
| NANOGP8 |  |  |  |  |  |
| HIPK2 |  |  |  |  |  |
| TFAP4 |  |  |  |  |  |
| SENP1 |  |  |  |  |  |
| RCOR1 |  |  |  |  |  |
| CD274 |  |  |  |  |  |
| FLOT2 |  |  |  |  |  |
| CCN6 |  |  |  |  |  |
| CUX1 |  |  |  |  |  |
| TBX1 |  |  |  |  |  |
| NES |  |  |  |  |  |
| MIR23B |  |  |  |  |  |
| MIR29C |  |  |  |  |  |
| MIR203A |  |  |  |  |  |
| MIR155 |  |  |  |  |  |
| MIR370 |  |  |  |  |  |
| LINC00312 |  |  |  |  |  |
| ASAP1-IT1 |  |  |  |  |  |
| DUBR |  |  |  |  |  |
| MSC-AS1 |  |  |  |  |  |
| MIR193BHG |  |  |  |  |  |
| FAM225B |  |  |  |  |  |
| LINC00313 |  |  |  |  |  |
| FAM225A |  |  |  |  |  |
| SNHG18 |  |  |  |  |  |
| LINC02085 |  |  |  |  |  |
| MIR31HG |  |  |  |  |  |
| AGAP2-AS1 |  |  |  |  |  |
| SNHG12 |  |  |  |  |  |
| MIR9-3HG |  |  |  |  |  |
| LINC00239 |  |  |  |  |  |
| FAM201A |  |  |  |  |  |
| UNC5B-AS1 |  |  |  |  |  |
| PXN-AS1 |  |  |  |  |  |
| IGF2-AS |  |  |  |  |  |
| MIR141 |  |  |  |  |  |
| MIR200A |  |  |  |  |  |
| MIR200B |  |  |  |  |  |
| MIR200C |  |  |  |  |  |
| MIR429 |  |  |  |  |  |
| MIR542 |  |  |  |  |  |
| MIR30B |  |  |  |  |  |
| MIR30D |  |  |  |  |  |
| MIR30E |  |  |  |  |  |
| MIR130B |  |  |  |  |  |
| MIR192 |  |  |  |  |  |
| MIR193B |  |  |  |  |  |
| MIR215 |  |  |  |  |  |
| MIR17 |  |  |  |  |  |
| LINC02693 |  |  |  |  |  |
| C5ORF66 |  |  |  |  |  |
| MIR22HG |  |  |  |  |  |
| MEG3 |  |  |  |  |  |
| SCAMP1-AS1 |  |  |  |  |  |
| ASH1L-AS1 |  |  |  |  |  |
| LOC730101 |  |  |  |  |  |
| MIR600HG |  |  |  |  |  |
| NEAT1 |  |  |  |  |  |
| PAXIP1-AS1 |  |  |  |  |  |
| MALAT1 |  |  |  |  |  |
| MIR205 |  |  |  |  |  |
| MIR30A |  |  |  |  |  |
| MIR145 |  |  |  |  |  |
| SPRY4-IT1 |  |  |  |  |  |
| MIR21 |  |  |  |  |  |
| MIR300 |  |  |  |  |  |
| LINC-ROR |  |  |  |  |  |
| HOTAIR |  |  |  |  |  |
| MIR34A |  |  |  |  |  |
| TUG1 |  |  |  |  |  |
| MIR204 |  |  |  |  |  |
| MIR221 |  |  |  |  |  |
| MIR101-1 |  |  |  |  |  |
| MIR182 |  |  |  |  |  |
| MIR186 |  |  |  |  |  |
| MIR137 |  |  |  |  |  |
| ZEB2-AS1 |  |  |  |  |  |
| MIR373 |  |  |  |  |  |
| H19 |  |  |  |  |  |
| ZFAS1 |  |  |  |  |  |
| MIR506 |  |  |  |  |  |
| MIR1271 |  |  |  |  |  |
| MIR15B |  |  |  |  |  |
| MIR375 |  |  |  |  |  |
| MIR150 |  |  |  |  |  |
| MIR211 |  |  |  |  |  |
| MIR10B |  |  |  |  |  |
| MIR134 |  |  |  |  |  |
| MIR138-1 |  |  |  |  |  |
| MIR183 |  |  |  |  |  |
| MIR30C1 |  |  |  |  |  |
| SNHG6 |  |  |  |  |  |
| MIR616 |  |  |  |  |  |
| CCAT2 |  |  |  |  |  |
| MIR187 |  |  |  |  |  |
| HOXA11-AS |  |  |  |  |  |
| MIR153-1 |  |  |  |  |  |
| MIR491 |  |  |  |  |  |
| GHET1 |  |  |  |  |  |
| MIR148A |  |  |  |  |  |
| MIR106B |  |  |  |  |  |
| MIR655 |  |  |  |  |  |
| MIR9-1 |  |  |  |  |  |
| CPS1-IT1 |  |  |  |  |  |
| MIR26B |  |  |  |  |  |
| MIR124-1 |  |  |  |  |  |
| MIR9-3 |  |  |  |  |  |
| MIR96 |  |  |  |  |  |
| MIR489 |  |  |  |  |  |
| MIR379 |  |  |  |  |  |
| HOTTIP |  |  |  |  |  |
| MIR15A |  |  |  |  |  |
| MIR194-1 |  |  |  |  |  |
| NKILA |  |  |  |  |  |
| MIR630 |  |  |  |  |  |
| CCAT1 |  |  |  |  |  |
| MIR143 |  |  |  |  |  |
| MIR132 |  |  |  |  |  |
| MIR644A |  |  |  |  |  |
| MIR361 |  |  |  |  |  |
| MIR490 |  |  |  |  |  |
| UCA1 |  |  |  |  |  |
| MIR382 |  |  |  |  |  |
| PVT1 |  |  |  |  |  |
| MIR23A |  |  |  |  |  |
| MIR485 |  |  |  |  |  |
| MIR31 |  |  |  |  |  |
| MIR125B2 |  |  |  |  |  |
| AFAP1-AS1 |  |  |  |  |  |
| MIR598 |  |  |  |  |  |
| MIR876 |  |  |  |  |  |
| MIR181B1 |  |  |  |  |  |
| MIR30C2 |  |  |  |  |  |
| MIR1236 |  |  |  |  |  |
| MIR612 |  |  |  |  |  |
| MIR139 |  |  |  |  |  |
| MIR22 |  |  |  |  |  |
| MIR520C |  |  |  |  |  |
| MIR495 |  |  |  |  |  |
| MIR29B1 |  |  |  |  |  |
| SNHG15 |  |  |  |  |  |
| HOXA-AS2 |  |  |  |  |  |
| ZEB1-AS1 |  |  |  |  |  |
| CBR3-AS1 |  |  |  |  |  |
| MIR195 |  |  |  |  |  |
| MIR27B |  |  |  |  |  |
| NORAD |  |  |  |  |  |
| BCAR4 |  |  |  |  |  |
| CYTOR |  |  |  |  |  |
| MIR214 |  |  |  |  |  |
| MIR340 |  |  |  |  |  |
| MIR661 |  |  |  |  |  |
| MIR9-2 |  |  |  |  |  |
| MIR26A1 |  |  |  |  |  |
| MIR16-2 |  |  |  |  |  |
| MIR100 |  |  |  |  |  |
| LINC00261 |  |  |  |  |  |
| LINC01186 |  |  |  |  |  |
| LINC02231 |  |  |  |  |  |
| LINC02599 |  |  |  |  |  |
| BX111 |  |  |  |  |  |
| TBILA |  |  |  |  |  |
| SLC25A25-AS1 |  |  |  |  |  |
| LOC111162621 |  |  |  |  |  |
| TP73-AS1 |  |  |  |  |  |
| MIR93 |  |  |  |  |  |
| MIR153-2 |  |  |  |  |  |
| MIR448 |  |  |  |  |  |
| MIR338 |  |  |  |  |  |
| HULC |  |  |  |  |  |
| MIR1290 |  |  |  |  |  |
| PANDAR |  |  |  |  |  |
| MIR135B |  |  |  |  |  |
| MIR10A |  |  |  |  |  |
| LET-7A-5P |  |  |  |  |  |
| MIR1 |  |  |  |  |  |
| MIR103A-3P |  |  |  |  |  |
| MIR122-5P |  |  |  |  |  |
| MIR124-3P |  |  |  |  |  |
| MIR128A |  |  |  |  |  |
| MIR128-2 |  |  |  |  |  |
| MIR129 |  |  |  |  |  |
| MIR133A |  |  |  |  |  |
| MIR140-3P |  |  |  |  |  |
| MIR142-3P |  |  |  |  |  |
| MIR16-5P |  |  |  |  |  |
| MIR181A |  |  |  |  |  |
| MIR181C |  |  |  |  |  |
| MIR181D |  |  |  |  |  |
| MIR18A |  |  |  |  |  |
| MIR18B |  |  |  |  |  |
| MIR197 |  |  |  |  |  |
| MIR199A |  |  |  |  |  |
| MIR19B-3P |  |  |  |  |  |
| MIR210 |  |  |  |  |  |
| MIR222 |  |  |  |  |  |
| MIR223 |  |  |  |  |  |
| MIR224 |  |  |  |  |  |
| MIR24-1 |  |  |  |  |  |
| MIR27A |  |  |  |  |  |
| MIR296-5P |  |  |  |  |  |
| MIR301 |  |  |  |  |  |
| MIR30C-5P |  |  |  |  |  |
| MIR324-3P |  |  |  |  |  |
| MIR328-3P |  |  |  |  |  |
| MIR335-5P |  |  |  |  |  |
| MIR33A |  |  |  |  |  |
| MIR34B |  |  |  |  |  |
| MIR34C |  |  |  |  |  |
| MIR374A-5P |  |  |  |  |  |
| MIR486-3P |  |  |  |  |  |
| MIR488 |  |  |  |  |  |
| MIR513 |  |  |  |  |  |
| MIR518F |  |  |  |  |  |
| MIR628-5P |  |  |  |  |  |
| MIR636 |  |  |  |  |  |
| MIR769-5P |  |  |  |  |  |
| MIR92A-3P |  |  |  |  |  |
| MIR95 |  |  |  |  |  |
| MIR98 |  |  |  |  |  |
| MIR99A |  |  |  |  |  |
| MIR291A-5P |  |  |  |  |  |
| MIR3092-3P |  |  |  |  |  |
| MIR3104-3P |  |  |  |  |  |
| MIR422B |  |  |  |  |  |
| MIR5046 |  |  |  |  |  |
| MIR669H-3P |  |  |  |  |  |
| MIR674-5P |  |  |  |  |  |

Supplementary Figure1：EMT differentially expressed genes were connected to analysis the overall survival of HCC patients.
